# 3D Concrete Printing: A Systematic Review of Rheology, Mix Designs, Mechanical, Microstructural, and Durability Characteristics

**DOI:** 10.3390/ma14143800

**Published:** 2021-07-07

**Authors:** Atta Ur Rehman, Jung-Hoon Kim

**Affiliations:** Construction Robot and Automation Laboratory, Department of Civil and Environmental Engineering, Yonsei University, 50 Yonsei-ro, Seodaemun-gu, Seoul 03722, Korea; attabrcian@gmail.com

**Keywords:** concrete 3D printing, extrusion, printable concrete, rheology, mechanical properties, compressive strength, flexural strength, tensile strength, anisotropy, printing process parameters, microstructure, durability

## Abstract

This paper provides a state-of-the-art report on the up-to-date research on the emerging 3D concrete printing technology from the concrete materials perspective. It reviews the recent research focused on understanding and characterizing the rheological necessities of the concrete printing process and discusses how the researchers are tailoring compatible mix proportions for the 3D concrete printing process by using eco-friendly binders, waste aggregates, chemical admixtures, and nano-additives. This paper systematically evaluates anisotropic behavior in the mechanical properties of printed concrete and establishes an order for anisotropic behavior in the compressive, flexural, and tensile strengths along three different axes (*X*, *Y*, and *Z* axes) of printed concrete. It evaluates the ratio of flexural strength to the compressive strength of printed concrete along the above three axes. This article explains the influence of variation of printing process parameters on the mechanical properties and discusses reinforcement approaches used for increasing structural performance. The microstructure at the interface of adjacent layers and also at the interface of the reinforcement-cement matrix is discussed. The recent research on the durability performance of printed concrete is critically discussed and future research needs for 3D concrete printing are identified in this paper.

## 1. Introduction

**Figure 1:**
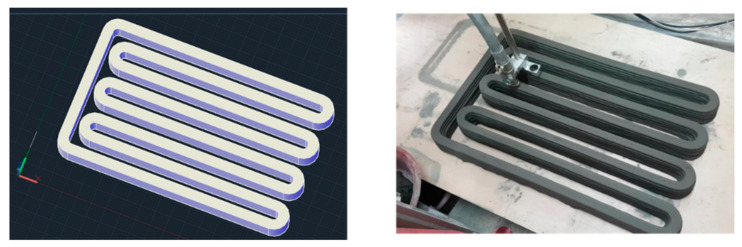
Three-dimensional model in CAD environment and concrete extrusion along the print path.

Concrete 3D printing is an emerging technique for the construction of buildings and infrastructure. In this method, a 3D model of an object is created in computer-aided design (CAD) software, which is then divided into slices, and a G-code is generated for the movement of the print head to extrude the concrete. The extrusion of concrete through the nozzle and movement of the print head are computer-controlled [1,2]. Concrete used in 3D printing is passed through the stages of pumping, extrusion through the nozzle, and accurate deposition in the successive layers to achieve a three-dimensional object, as shown in Figure 1. Extruded concrete supports self-weight and retains its shape without the use of formwork and bonds with the subsequent layers [3,4,5]. This layer-by-layer concrete extrusion is also called concrete additive manufacturing and concrete ink printing.

The application of 3D concrete printing technology in construction is considered a new period for the industry due to its potential to disrupt conventional construction practices [6,7]. It is receiving enormous recognition due to its unique characteristics, such as construction without the use of formwork, reduced human involvement, minimum material wastage, and mass customization [8,9]. This technology completely saves the formwork costs, reduces the labor cost by 50–80%, and decreases the wastage of construction materials at the site by 30–60% [10]. Additionally, due to an increase in productivity at the construction site and a reduction in the construction time, it has the potential to further reduce construction costs [11]. It is presumed as a promising technique for the construction of structural-space habitats at the moon and Mars [12,13,14,15,16,17] and also for military purposes [18]. Its promised freedom of design can enable architects and engineers to manufacture complex shapes and aesthetically appealing concrete elements and facades [1,19]. Numerous concrete structures have been constructed using concrete 3D printing technology, such as two-story municipality building in Dubai by Apis Cor [20], community village in Austin by ICON [21], vehicle-hiding concrete arches in California by U.S. Marines Corps [22], houses by COBOD and Peri Group in Berlin [23], river revetment wall in Suzhou by Winsun [24], prestressed bicycle bridge at Eindhoven [25], the pedestrian arch bridge at Shanghai [26], and optimized-reinforced concrete beams at Ghent and Naples [27,28]. However, despite such diverse showcase construction projects, the use of concrete 3D printing for routine constructions is uncommon due to the technical challenges. At present, the understanding of the rheological requirements, mechanical and durability performance of 3D concrete printing is at the developing stages. Conventional and high-performance concrete mixes cannot be directly used for concrete 3D printing construction due to the incompatibility of their rheological characteristics with the requirements of the printing process at different stages. Therefore, researchers are rigorously investigating alternative concrete mixes for fulfilling the transporting and stiffening requirements of the printing process.

This paper aims to provide a systematic review of the fresh performance and rheological test systems of 3DCP, and the influence of concrete ingredients on the rheology of printable concrete. It can help researchers and engineers to measure the printability performance of their mixes and tune the rheology of the concrete according to their printing requirements. The critical review section of mechanical properties aims to quantify the anisotropic pattern in printed concrete and draw a relationship between flexural and compressive strengths that can be helpful to structural engineers for designing safe, printed concrete structures. The microstructure and durability sections discuss the occurrence of higher porosity and cracks in printed concrete and the expected influence of these strains on the long-term service life of printed concrete. Additionally, future research needs are understanding the early stage, hardened, and long-term performance of extruded concrete are also reflected in this review.

This paper has been organized into 10 sections in such a way that it fulfills the needs of beginners as well as advanced researchers in this field. Section 2 introduces the rheology of printable concrete mixes, and Section 3 discusses the stages of the printing process and rheological needs at each of the steps that a potential concrete mix has to satisfy for use in printing. Test methods for measuring the concrete conformability with the printing process requirements are also scrutinized. Section 4 presents printable mixes as well as research attempts to regulate the rheology of printable concrete by using eco-friendly binders, aggregates, chemical admixtures, and nanomaterials. Section 5 evaluates anisotropy in the mechanical properties of printed concrete, and Section 6 discusses the effect of the printing process parameters upon the mechanical properties. Section 7 covers different reinforcement strategies used for increasing the structural application of printed concrete. Section 8 and Section 9 provide a comprehension of microstructural and durability properties of printed concrete, respectively. Section 10 presents concluding remarks of this comprehensive review study and identifies future research needs for concrete 3D printing.

## 2. Rheology

Bingham model has been widely used to characterize the flow of 3D-printable concrete [29,30,31,32,33], which is given below

(1) $$\tau =\tau_{0}+\mu \gamma^{.}$$

τ and *γ˙* represent shear stress and shear rate, respectively, whereas *τ*_0_ and *μ* represent yield stress and plastic viscosity of concrete, respectively [34]. Three-dimensionally printable concrete is a semi-solid material; it flows when certain shear stress is applied upon it. Static yield stress is the peak shear stress required to initiate the flow in the static concrete. Dynamic yield stress is the shear stress needed to maintain the flow once concrete starts to flow from the rest position. If the external shear force is removed, concrete will stop flowing, flocculation of the particles start due to the interparticle interaction, and static yield stress is restored. This phenomenon is called thixotropy [35]. As time goes on, the static yield stress of fresh concrete mix increases due to interparticle interaction and the start of the cement hydration, and this process is called structuration [36,37]. The structuration rate (A_thix_) represents the rate of increase in static yield stress with time [38]. Dynamic yield stress and plastic viscosity influence concrete pumping and extrusion stages, whereas static yield stress, thixotropy, and structuration rate define shape retention and buildability after the extrusion [3]. These stages of concrete printing will be discussed in detail in Section 3. Static yield stress is measured with stress growth test, whereas dynamic yield stress and plastic viscosity are measured with flow curve test. Both tests are conducted using a concrete rheometer. The stress growth test involves the rotation of a shear vane at a fixed low speed (0.025 rev/s) in a concrete bucket and the measurement of the corresponding torque [39]. In this test, the torque initially increases without the significant flow of the concrete but then at a certain value of the torque, the flow of the concrete increases, which corresponds to the static yield stress. Hand-held shear vane has also been used for the measurement of static yield stress [40,41], which is a popular test method in soil mechanics [42]. Flow curve test is used to calculate dynamic yield stress and plastic viscosity values of the concrete, which involves two stages: initial breakdown period to remove the thixotropic structure of the concrete and then reduction in the speed of the shear vane in incremental steps. For the breakdown period, the rheometer is rotated at its maximum speed whereas, at the second stage, speed is usually reduced in six steps that are maintained for a short time interval. Torque values are plotted against the rotation speed of the shear vane to obtain a flow curve plot [43]. Thixotropy can be measured by using the constant shear rate test [44,45], hysteresis loop test [46], and reflocculation test [36]. The structuration rate has been measured by conducting stress growth tests at different time intervals before setting the concrete [36,38].

**Figure 2:**
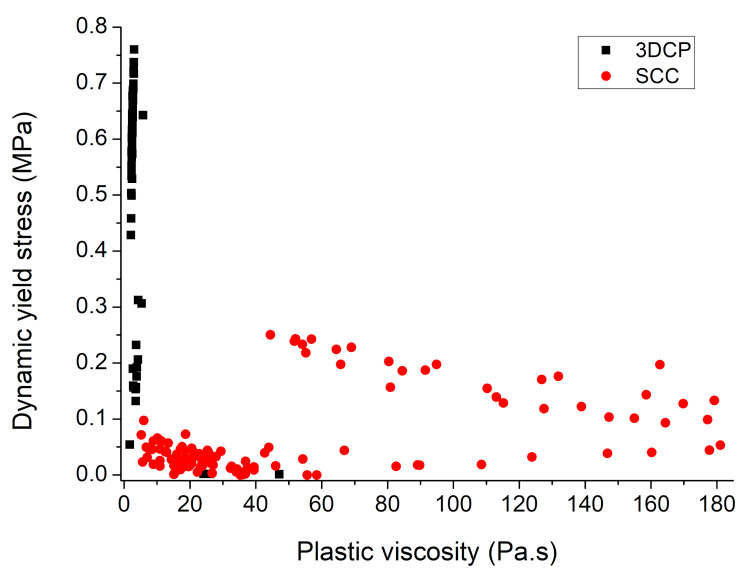
A comparison of rheographs of 3D-printable concrete and self-compacting concrete (SCC) [44,54,58,60,61,62,67,68,69,70,71,72].

The 3D concrete printing process requires contradicting rheological requirements. It needs high workability during the pumping phase before extrusion, but it needs low workability and high thixotropy after extrusion for better buildability of concrete [3,31,47,48]. During the concrete printing process, a balance is needed between the rheological requirements of pumping, extrusion, and buildability stages [49,50,51]. If concrete material with low yield stress is used to help pumping and extrusion stages, then extruded concrete will not retain its shape. On the contrary, if stiff concrete with high yield stress and viscosity is used for attaining shape retention and high buildability, then it will increase the required pumping power, and extrusion through the nozzle will be difficult [49]. Table 1 summarizes the range of static yield stress, and Table 2 summarizes the range of dynamic yield stress and plastic viscosity values for various printable concrete mixes reported in the literature [29,36,38,40,44,52,53,54,55,56,57,58,59,60,61,62]. Table 1 shows that reported values of static yield stress for printable mixes lie as low as 0.16 kPa for calcium sulfoaluminate cement paste ink [54] and as high as 6.8 kPa for stiff mixes of nano-clay containing high volume fly ash mortar [56]. Table 2 shows that most of the reported values of dynamic yield stress lie within the range of 0.05–0.76 kPa, whereas most of the values of plastic viscosity lie within the range of 1.6–5.8 Pa·s with few exceptions. Research studies that have simultaneously measured static yield stress, dynamic yield stress, and plastic viscosity for a printable concrete mix proportion are rare in the existing literature. Table 1 and Table 2 indicate that there is no absolute value of static yield stress, dynamic yield stress, and plastic viscosity values for printable concrete [63,64]. Even rheological values of the same concrete mix proportion measured with two different kinds of rheometers can vary. Additionally, numerous types of concrete printers with different specifications and capabilities are currently in use at research laboratories and construction sites [65]. Hence, a single mix designed for one concrete printer may not be printable with a different concrete printer due to the reduced capacity of its pump, different pipe length, and diameter as well as nozzle dimensions. Figure 2 shows a comparison of rheographs of 3D-printable concrete with self-compacting concrete (SCC), which is also a rheology-sensitive concrete type. SCC mixes have a low yield stress to facilitate the self-leveling and passing through the dense reinforcement but have high viscosity to resist segregation during flow. Three-dimensionally printable mixes do not contain coarse aggregate and the susceptibility of these mixes to segregate is comparatively low but these mixes have to retain their shape after extrusion, therefore, printable concrete mixes are designed for low viscosity and high yield stress. It is worth noticing that SCC mixes have high formwork pressure, but printable mixes have the capability to bear self-weight and hence do not need formwork.

The rheological properties of 3D concrete printing reported in the previous literature have been mostly measured without feeding the concrete in the 3D concrete printer. However, concrete is subjected to excessive shearing when pumped through a pipe to the nozzle of the printer, and this process changes the concrete rheology. In addition, the extrusion through the narrow nozzle alters the composition and rheology of concrete. These changes in concrete are dependent upon the material properties such as plastic viscosity and dynamic yield stress of concrete, plastic viscosity of lubrication layer, and 3D printer associated properties such as pumping rate, pipe radius, and geometry of the nozzle [73]. Characterization of the lubrication layer due to changes in mix design, pumping rate, pipe diameter, and nozzle geometry are open research questions. Different thixotropic behavior and structuration rates have been reported in the literature. Kruger et al. used the ICAR rheometer and reported an average structuration rate of 43.2 Pa/min [55]. Keita et al. reported 27, 15, and 12 Pa/min for mix proportions with w/c ratios of 0.40, 0.35, and 0.20, respectively [74]. The structuration rate should not exceed a certain limit; otherwise, the open time for printing operation will be very low [53,60]. Additionally, cold joints can be formed between adjacent layers due to rapid structuration and high printing time gaps [75,76,77,78,79]. The formation of cold joints reduces the bond strength between layers [49,78,80].

## 3. Printing Process Requirements

Concrete used in 3D printing is passed through the stages of pumping, extrusion, and layer-by-layer deposition. These stages require special characteristics from a concrete mix to conform with the sophisticated needs of the printing process, which are discussed in detail in the following subsections.

### 3.1. Pumpability

Pumpability is the capability of a mix to be transported through a pipe under pressure. Progressive cavity pumps, positive displacement pumps, and ram extruders have been usually used to transport concrete from the mixing tank or hopper to the extruding nozzles [53,81,82,83]. If concrete pumping is stopped for a short time, then the structuration of concrete occurs within the hose of the printer and consequently increases the pressure required to resume pumping. Concrete mix design affects its pumpability performance during 3D printing. Mohan et al. [84] varied the aggregate-binder ratio from 1.0 to 1.4 and 1.8 and observed that pumping requirements increased from 9 to 12 bar and 17 bar, respectively, which shows that increasing the aggregate content increases the pressure required for pumping printable concrete. Concrete is a heterogeneous material. When printable concrete is pumped through a pipe, the flow of concrete is divided into two layers as lubrication layer and bulk concrete due to the particle migration. The rheology of both layers is different, and the pumping requirement for a printable concrete mix depends on the combined yield stress and plastic viscosity of both layers. Tribometers and viscometers can be used for the measurement of the rheology of the lubrication layer [63]. Mohan et al. [85] observed that plastic viscosity and yield stress of the lubrication layer produced due to pumping of printable concrete are dependent upon the aggregate-binder ratio. Matthäus et al. [86] observed that the addition of limestone decreases the pressure required for pumping lightweight concrete. Sooryanarayana et al. [87] observed that the vibration of concrete reduces its yield stress and facilitates the pumping of concrete. The integration of such a vibrating system with the hose and nozzle of a 3D printer may allow the use of coarse aggregate in printing concrete. Stiff mixes need higher vibrational velocity, and fluid mixes need a lower value of critical vibrational velocity for reducing extrusion pressure [88]. Research on the pumpability of various printable concrete types, the consequence of pumping on their rheology, and test methods for measurement of the pumpability are limited. Further research is needed in this regard.

### 3.2. Extrudability

Extrudability is the capability of concrete to pass under pressure through the contracting nozzle without segregation and produce a continuous filament. Extrusion of concrete through the nozzle is an essential part of the 3D concrete printing process. Le et al. [40] observed that mix proportions with high sand content segregated during extrusion and blocked the pipe. Le et al. [40] also observed that concrete mixes became unextrudable when the yield stress of concrete exceeded a certain value (0.9 kPa) due to the structuration. Nerella et al. [59] observed that increased fineness of solid materials at the fixed water amount decreases extrudability. It was also noted that extrudability cannot be quantified from the simple flow tests of concrete, but rheological parameters can provide an indication of concrete extrudability. Panda et al. [53] observed the occurrence of segregation in geopolymer concrete at the extrusion stage when the sand-binder ratio increased from 1.1 to 1.9. Le et al. [40] presented a test method for the extrudability of concrete by printing filaments of 9 mm width and 300 mm length. A concrete mix that extruded test-filament length without any segregation in the nozzle and breakage was considered as a successful mix proportion for the extrusion stage. Researchers at Technical University Dresden developed a different extrudability test. This test measures the energy consumed for extruding the concrete at a fixed flow rate [59]. Stiff mixes are difficult to extrude, filaments rupture while printing stiff concrete, and voids are created. These voids would have a significant effect on flexural strength after the hardening of concrete [60]. Esnault et al. [89] introduced a different approach to reducing the extrudability requirements of concrete. Their method involves the use of fluid concrete to reduce the pumping pressure requirements and the addition of appropriate accelerator dosage at the nozzle part of the printer to accelerate the structuration of concrete and enable it to sustain self-load. The above discussion shows that the extrudability of concrete is dependent upon the following factors:

- Mix proportion (water-binder ratio, aggregate-binder ratio, aggregate characteristics, chemical admixture dosage);
- Rheological properties of concrete;
- 3D printer (pumping power, pipe length and diameter, inlet and outlet dimensions of the nozzle).

### 3.3. Shape Retention

Shape retention is the capability of the extruded concrete layer to retain its cross-section equal to the cross-section of the nozzle. The shape retention factor for printed concrete can be measured by dividing the width of the printed layer by the width of the printer nozzle, as given below [90].

(2) $$S=\frac{W_{f}}{W_{n}}$$

Here, S shows the shape retention factor, and *W_f_* and *W_n_* are the width of the extruded filament and the nozzle, respectively. Chougan et al. [91] tested the shape retention capability by first printing six layers of concrete and then measured the height of each layer after one hour. As this new shape retention test focuses on the height of multiple layers instead of one layer, therefore it gives a more accurate assessment of the shape retention capability of a potential concrete mix after extrusion. Shape retention can be affected by the printing speed. Lee et al. [92] tested the effect of different printing speeds (5, 7.5, 10, and 12.5 cm/s) upon the width of the printed filament and observed that as the printing speed increased from 5 to 7.5 cm/s, 10 cm/s, and 12.5 cm/s, and the width of the filament decreased by 28%, 42%, and 51%, correspondingly. The shape retention capability of concrete can be increased by the addition of nano-additives [36,91,93,94,95,96,97,98].

**Figure 3:**
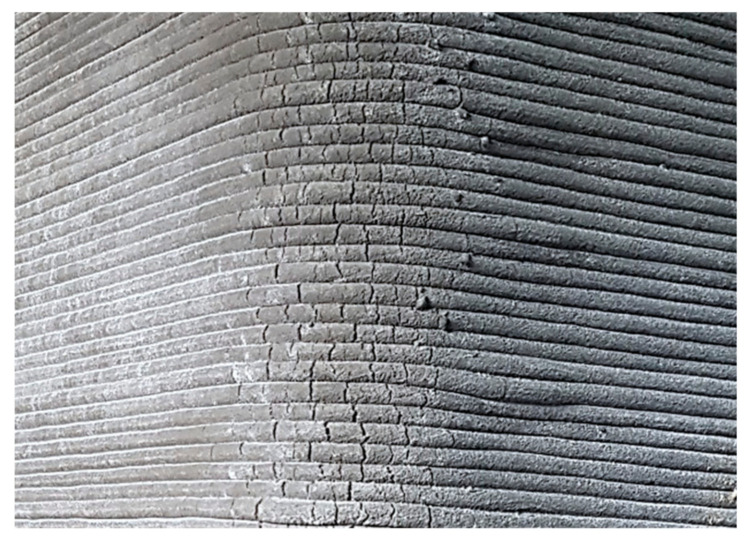
Development of cracks at the corner of a fresh-printed concrete wall.

Printing the curved parts of a concrete layer with a rotating circular or rectangular-shaped nozzle extrudes a larger amount of concrete at the corners. This results in increased width of the printed layers at the curves compared to its width at the straight parts. More concrete is deposited on the inner side at the corners compared to the outer side during the rotation of the nozzle. Tearing and cracking of the printed-curved layers can happen on the outer side soon after the deposition, and these cracks can expand due to the weight of the successively deposited layers at earlier ages, which will result in poor surface finishing, as shown in Figure 3. Consequently, reduced mechanical performance could be observed at the curved sections of printed elements. Liu et al. [99] investigated the non-uniform concrete distribution at the corners with a rotational rectangular nozzle. Results indicated that a lower aspect ratio of the nozzle and a higher corner radius of curvature help in maintaining the uniform width at the corners. However, limiting the print path to the higher radius of curvature can limit the promised geometrical freedom of 3DCP.

### 3.4. Printing Open Time

It is the time period after mixing water, cement, aggregate, and other additives during which concrete is extrudable through the nozzle. The printing open time of a candidate concrete mix must be greater than the time required for extruding the concrete during the printing operation. Printing open time is dependent upon mix design [53,60], and it can be regulated according to the needs of the printing process by using retarders or accelerators [40]. The expiration of the printing open time has been associated with the evolution of the yield stress to a stage beyond which concrete does not remain extrudable [40,53,100]. Chen et al. [90] measured the printing open time of limestone-calcined clay-based printable cementitious mixes by printing multiple concrete filaments of 80 cm length and 4 cm width with a time gap of 10 min. Extrusion rate and nozzle speed were fixed, and concrete was pre-sheared before pumping to break down any structuration. The time when concrete filaments started tearing after extrusion indicated the expiration of the printing open time.

### 3.5. Buildability

Buildability is the characteristic of a concrete mix proportion to be successively printed up to a specific height without significant deformation or collapse of the layers. It depends upon the rheological properties of concrete, printing process parameters, and geometry of the target concrete structure. Researchers have presented numerous methods for the evaluation of the buildability of concrete. There is no consensus upon a standardized experimental test method for the measurement of concrete buildability. Le et al. [40] were the first to measure the buildability of concrete by counting the number of layers that can be printed over the bottom layer. Kazemian et al. [101] proposed layer settlement and cylinder stability test methods for the measurement of the buildability of concrete. In the layer settlement test, a layer of concrete is printed first, and then after a specific time gap, another layer is printed over the first layer, the deformations in the first layer are measured. The cylinder stability test evaluates the deformations in cylindrical concrete specimens under incrementally added loads. Zhang et al. [102] printed a hollow square (30 cm × 30 cm) with different concrete mixes and measured the buildability by calculating the maximum height that could be printed with each mix proportion. The early age strength of concrete is called green strength, which is due to the combined result of interparticle friction and cohesion [98]. Yuan et al. [103] calculated the buildability of concrete by measuring the green strength and deformation of concrete after applying incremental loads with specified time gaps. If the deformation of concrete was less than 0.2% of total height, the buildability was considered acceptable. Perrot et al. presented an analytical model to measure the buildability of 3DCP by comparing the green strength of the bottom layer with the incremental increase in weight due to the printing of layers over the bottom layer. This buildability model suggests a safe construction rate and it can forecast the failure time if the critical construction rate is exceeded during a printing process.

(3) $$t_{f}=\frac{\tau_{0,0}}{\frac{\rho gR}{\alpha_{geom}}-A_{thix}}$$

Here, *t_f_* is critical failure time, *τ*_0,0_ is the initial yield stress of concrete, *ρ* is density, *g* is the gravitational force, *R* is the rate of construction, *α_geom_* is a geometrical factor and depends on the shape of extruded filament, *A_thix_* represents the structuration rate [38]. Wolf et al. [83] considered the concrete green strength at initial stages (0–90 min after concrete extrusion) similar to that of cohesive soil and developed a buildability model using the Mohr-coulomb concept and correlated the shear strength of 3D-printable concrete with interparticle cohesion, angle of friction and normal stress. The printing process of a hollow cylindrical object was modeled in Abaqus using material properties obtained with experimental tests. The hollow cylinder was also printed with a 3D printer for validation of the numerical model. The printing process showed that the developed model slightly overrates the strength and stability of the 3D-printable concrete. The overestimation is attributed to the compaction of concrete, use of larger specimens, negligence of printing parameters, and effect of the geometry of printed filament in the model [104]. During the 3D printing of an object, two failure modes are expected. It may fail due to the plastic yielding of concrete, or it may become unstable due to elastic buckling of printed height. The failure mode is affected by target object geometry, yield stress, structuration rate as well as vertical construction rate. Panda et al. observed that elastic deformation can happen due to the low stiffness of concrete, whereas plastic failure may happen due to inadequate green strength [98]. Jayathilakage et al. [105] reported that using a test specimen with a higher aspect ratio such as 2, as used in the work of [98,104], may not be suitable for 3DCP because extruded concrete layers have lower aspect ratios. Therefore, the authors emphasized the use of a test specimen with a lower aspect ratio for predicting the failure of concrete during concrete 3D printing. Suiker [106] developed a buildability model to predict which failure mode (plastic collapse or elastic buckling) occurs first while printing a structure. Model results were compared with the printing of a straight wall, which showed that the mechanistic model slightly underestimates the actual concrete buildability. Kruger et al. [107] presented a bi-linear thixotropy model for the measurement of the buildability of 3D concrete printing based on the reflocculation rate, structuration rate, and the static and dynamic yield stress of concrete. In another study, Kruger et al. [55] presented a constructability model to assess the optimum print speed and layer height for constructing a specified vertical height within a defined time span. This model is based on the evolution of the rheological parameters with time and self-weight of concrete and accounts for the plastic failure of concrete. Reiter et al. [108] reported that in order to avoid failure due to self-weight, the strength of fresh concrete should evolve linearly with time, whereas to avoid buckling failure, the strength of concrete should evolve with the third power after extrusion. Rieter et al. proposed controlled structuration of concrete by the combined use of retarders and accelerators to achieve maximized buildability for concrete.

Muthukrishnan et al. [109,110] studied microwave heating of geopolymer concrete as a technique to gain a set on demand. Geopolymer concrete was heated for 5, 10, and 20 s in a microwave oven (power = 1200 W, frequency = 2.45 GHz), and its effects on fresh properties were measured. Microwave heating increased the structuration rate and buildability of concrete. Results also showed that 10 s is the best time for the microwave heating of geopolymer, which increased interlayer bond strength up to 122–127% compared to unheated specimens. Bhattacherjee and Santhanam [111] introduced the idea of spraying a high dosage of alkali-free aluminum sulfate accelerator (>8%) on the surface of printed filament to increase its stiffness and green strength. A 20 cm tall rectangular structure was printed, and the effects of the spraying accelerator on the buildability were observed. The height of the rectangular structure without the accelerator was 18.2 cm due to the deformation in layers under load, whereas the use of the accelerator provided 19.7 cm height for the rectangular section.

## 4. Printable Mix Designs and Influence of Concrete Ingredients on Rheology

**Figure 4:**
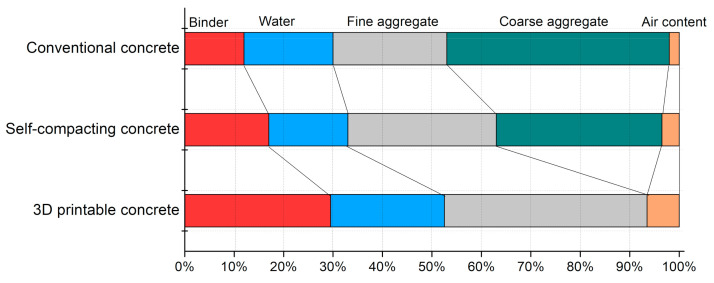
An illustrative comparison of percentage of materials (by volume) used in conventional concrete, self-compacting concrete, and 3D-printable concrete.

**Figure 5:**
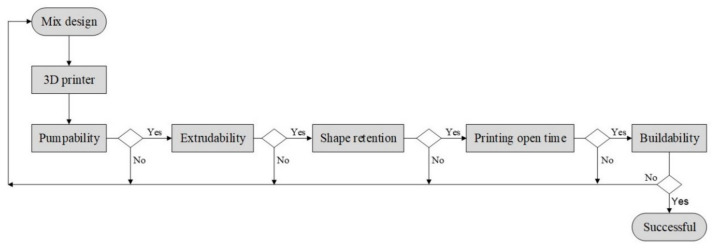
Iterative process of preparing a printable concrete mix.

Figure 4 presents an illustrative comparison of materials proportion in 3D-printable concrete, self-compacting concrete, and traditional concrete mixes. Three-dimensionally printable mixes are designed with higher content of binder and fine aggregate than conventional concrete mixes and SCC to increase its yield stress and shape retention capability. The design of a printable concrete mix is an iterative process. The compatibility of a concrete mix with the needs of the printing process (pumping, extrusion, shape retention, open time, buildability) is checked in sequential steps, as shown in Figure 5. If a concrete mix is found compatible with the former requirement, then it is tested for the succeeding requirement. Otherwise, mix design is changed by varying the ingredients, and again, its performance is observed. This process is continued until the concrete mix matches all the requirements of the printing operation [101]. Printable concrete mixes usually have low dynamic yield stress to help pumping and extrusion phases but have high thixotropic behavior after extrusion to increase static yield stress enabling the concrete to support the self-weight and weight of the subsequent printed layers [36,45,53,57,59]. Table 3 summarizes optimum printable mixes reported in the literature, which shows that printable mixes can be developed with the different binders and fine aggregate types, different water-binder ratios, sand-binder ratios, chemical admixture, and other additives such as fibers, nanomaterials, and clays. Most of the developed printable mix designs do not contain coarse aggregate to avoid blockage during pumping and extrusion stages. The eccentric screw pump is generally adopted as an extruder in 3DCP, and its components, rotor, and stator can only allow limited grain sizes. Higher amounts of the binder used in the production of printable concrete mixes raise questions about the environmental friendliness of this technology [112], but researchers are attempting to decrease its carbon footprints by adopting eco-friendly binders and recycled aggregates in mix designs, as discussed in Section 4.1 and Section 4.2 Printable mix proportions are intended to be stiff and display higher green strength to sustain the weight of the successive layers without plastic collapse. Therefore, a lower water-binder ratio and higher binder-sand ratio are used. Rheology of concrete ink is further tuned for printability and vertical constructability by using a high dosage of chemical admixtures such as viscosity modifying admixtures, superplasticizers, accelerators, and nanomaterials [113,114,115]. The size of the fine aggregate in most of the mixes lies below 2 mm. Some studies have used special concrete types such as foam concrete [116,117], underwater printable concrete [118], geopolymer concrete [119,120], magnesium potassium phosphate cement concrete [121,122], engineered cementitious composite [123], and earth-based materials [124]. The frequent reported water-binder ratio lies within the range of 0.30–0.40, and the frequent sand-binder ratio lies within the range of 1.2–2.0. Researchers have also developed printable mixes with a high dosage of fibers to reinforce concrete [61,125,126,127,128]. Additional research is needed for optimizing fiber-reinforced printable concrete mixes such as engineered cementitious composites and ultra-high-performance fiber-reinforced concrete as the addition of fibers reduces the workability and has negative consequences upon the performance of concrete during pumping and extrusion steps.

Researchers have used different concrete ingredients (eco-friendly binders, aggregates, chemical admixtures, and nanomaterials) in their mix designs to understand their effect upon the rheology and consequences upon the printing performance of the concrete. These research efforts have been discussed in the following subsections and summarized in Table 4.

### 4.1. Eco-Friendly Binders

The higher content of cement used in 3D concrete printing raises the sustainability issue of this technology [112]. Therefore, there is a great emphasis on replacing the cement with eco-friendly binders in 3D printing construction. Numerous researchers have attempted to develop printable mixes with eco-friendly binders and have investigated their effect on the rheology of concrete, as shown in Table 4. Chen et al. [145] studied the effect of three different grades of calcined clay (high, medium, low) on the rheology and extrudability of concrete. Metakaolin content was higher in high-grade calcined clay, followed by medium and then low-grade calcined clay. Results showed that the addition of calcined clay with higher metakaolin content reduced the workability and setting time of concrete and increased the extrusion pressure. It increased the compressive strength at an early age (0–4 h). Another research study reported improvement in the thixotropic behavior of 3D-printable concrete with the addition of metakaolin [54]. Panda and Tan [146] observed that the addition of silica fume increases the yield stress and structural buildability of concrete due to its finer particle size distribution and increase in the packing density of fresh concrete. Zhang et al. [102] observed that the substitution of 2% of binders with silica fume increased the buildability of concrete by 117% compared to the control mix. Its addition also increased thixotropy and green strength. Kazemian et al. [101] observed that the addition of silica fume improves the surface quality of printed concrete. Alghamdi et al. [147] observed that the addition of limestone reduced workability and yield stress of concrete, 30% of limestone addition as a binder in fly ash-based geopolymer improved shape stability and buildability of concrete. Rehman et al. [41] used municipal solid waste incinerated (MSWI) bottom ash and fly ash in the development of printable concrete. Test results showed that the addition of MSWI fly ash produces favorable properties for the printing of concrete compared to the use of MSWI bottom ash and controlled concrete mix proportion. Replacement of cement with 10% MSWI fly ash was recommended for use in concrete printing as this dosage decreased the setting time, increased the shear strength and buildability of fresh concrete. Muthukrishnan et al. [148] used rice husk ash to replace 20% of cement in a printable mix. The presence of rice husk ash increased the compressive and shear strength of fresh concrete. Isothermal calorimetry tests showed that the heat due to early hydration reaction was 80% higher than the control mix at the age of 40 min due to the filler effect of rice husk ash. These preliminary research studies show the beneficial effects of eco-friendly binders, but more comprehensive studies are required to investigate the effect upon the overall concrete printing process and also sustainability achieved by using such binders.

### 4.2. Aggregate

Researchers have investigated the influence of aggregate characteristics upon the behavior of printable concrete. Zhang et al. [60] studied the effect of increased content of aggregate on the rheology of a high thixotropic concrete mix. Sand-binder ratio was varied as 0.6, 0.8, 1.0, 1.2, and 1.5. Plastic viscosity and yield stress were increased by 16.4% and 129.8% as the sand-binder ratio increased from 0.6 to 1.2, but it reduced thixotropy by 18%. Mohan et al. [84] observed that increasing the sand-binder ratio from 1.0 to 1.4 increases yield stress from 0.67 to 0.82 kPa and viscosity from 17.1 to 43.1 Pa·s. Researchers have used recycled aggregate, under-used solids successfully in 3D concrete printing and have observed positive results in terms of buildability [149]. Mine tailings, a waste residue from ores, have been studied as a substitute for sand in 3D-printable concrete [150]. Experimental test results have shown that 30% replacement of sand with mine tailings produces optimum buildability and mechanical properties [151]. Sambucci et al. [152] used powder and granules of recycled tire rubbers to replace fine sand in 3D-printable concrete and reported that the addition of recycled tire rubber reduces strength, but it increases the acoustic and thermal insulation, ductility and reduces porosity. Concrete containing rubber granules and powder can be used for printing lightweight bricks, pavements, and insulation panels. Rahul and Santhanam [136] developed a lightweight printable concrete by replacing sand with lightweight expanded clay aggregate. A total of 30% substitution of sand with lightweight expanded clay aggregate was observed to be suitable for extrudability and printability. Further increase in the amount of lightweight aggregate caused segregation during concrete extrusion. Cuevas et al. [153] produced lightweight printable concrete mixtures by using waste glass and expanded thermoplastic microspheres. The addition of waste glass aggregate resulted in reduced setting time at earlier ages and decreased thermal conductivity in hardened printed composites. In contrast, the addition of expanded thermoplastic microspheres increased plastic viscosity, shape retention, buildability. Ding et al. [154] replaced natural sand in printable concrete with 25% and 50% recycled sand and measured the green strength and modulus of elasticity of concrete. The use of recycled sand had an insignificant influence on mechanical properties up to the age of 90 min, but it increased compressive strength and modulus of elasticity after 90 min. Xiao et al. [155] replaced 25% of natural sand with recycled sand in concrete 3D printing and observed that the addition of recycled sand made the mortars stiff and also increased the yield stress from 1.89 to 1.94 kPa. Ting et al. [156] tested the replacement of river sand with equally graded recycled glass for use in 3D printing and observed the effects upon fresh properties. The addition of recycled glass increased the spread of concrete, reduced the static yield stress, and negatively affected the buildability of concrete. However, recycled aggregate can increase the porosity of printed concrete and reduce the flexural strength, which needs additional research [157]. Craveiro et al. [158] replaced 10% of fine aggregate in 3D concrete printing with cork to print lightweight concrete parts with enhanced thermo-mechanical characteristics. Results showed the potential of cork as a sand substitute in concrete for printing building components with better thermal insulation properties. Zareiyan and Khoshnevis [159] designed a concrete mix fulfilling the rheological properties of 3D-printable concrete and examined the outcome of aggregate size (3/32″, 3/16″, 1/4″, and 1/2″) on the mechanical properties of concrete. Test results showed that concrete mix proportions with smaller aggregate sizes improve compressive strength at the fresh stage. Sand particles of 20 mm size have been attempted for use in 3D printing, but print quality gets poor and the density of voids increases with the rise of sand particles size [160]. An enhanced particle packing with increased aggregate size may provide better results in terms of print quality.

### 4.3. Chemical Admixtures

A high dosage of chemical admixtures is used in concrete designed for extrusion and buildability purposes. Dorn et al. [161] observed that the setting time of printing concrete can be controlled with accelerators such as potassium carbonate (K_2_CO_3_), sodium carbonate (Na_2_CO_3_), calcium nitrate (Ca(NO_3_)_2_), and triethanolamine (TEA). The proper dosage of these accelerators can regulate the setting time within 5–150 min. The above admixtures affect the hydration of the binder and crystallinity of a few hydration products. Khalil et al. [134] studied calcium sulfoaluminate as a potential accelerator to regulate the printability of concrete. A concrete mix proportion with 7% CSA cement and 93% ordinary Portland cement (OPC) exhibited better extrudability and buildability and increased the yield stress by 17 and 30 times compared to the control mix at the age of 20 and 25 min. Chen et al. [162] used tartaric acid as a retarder to control the setting of sulfoaluminate cement (SAC) for concrete 3D printing. Dosages up to 0.30% of tartaric acid increased the setting time and printing open time of SAC. The addition of 0.25% tartaric acid reduced the yield stress and plastic viscosity by 16% and 2.5%, respectively. Qian and De Schutter [163] studied the effect of naphthalene sulfonate formaldehyde (NSF) and polycarboxylate ester (PCE) admixtures upon dynamic yield stress and viscosity of concrete. The addition of both superplasticizers decreased the dynamic yield stress and thixotropic behavior of concrete due to the adsorption upon binder particles and creating a dispersion. The effect of PCE was more prominent than NSF. Researchers also observed that NSF is compatible with viscosity modifying admixtures (VMA) such as nano-clays to achieve a high thixotropic concrete mix proportion with low dynamic yield stress. Hydroxypropyl methylcellulose has been used as a thickening agent in printable concrete mixes to increase shape retention after extrusion, prevent segregation and increase thixotropy [49]. Chen et al. [90,164] measured the effect of VMA on rheology, shape stability, buildability, printing open time, green strength, and hydration reaction. Test results showed that the addition of VMA increases the yield stress, plastic viscosity, green strength, and required extrusion pressure of concrete. An excessive dosage of VMA hinders the progress of cement hydration. Researchers observed that the concrete mixture with 0.24% of the binder weight as VMA showed optimum performance for concrete printability. Sun et al. [165] observed that sodium carboxymethyl starch improves the water retention capacity of slag-based geopolymer concrete and helps in the extrusion of concrete.

### 4.4. Nanomaterials

Researchers have used nanomaterials to modify the fresh behavior of concrete in favor of 3D concrete printing [166,167]. Chougan et al. reported an increase in yield stress, shape retention, and buildability of alkali-activated cementitious materials with the addition of nano-attapulgite clay particles. In their study, the substitution of 1% of alkali-activated printable materials with nano-attapulgite was recommended for optimum printability performance of active alkali materials [95]. Panda et al. [96] added 0.1–0.5% nano-attapulgite clay in printable mixes. Results showed that nano-attapulgite increases the static yield stress and buildability of concrete, but its effect on the viscosity is not prominent. Zhang et al. [102] observed that the replacement of cement with 2% nano-clay can increase the buildability of concrete by 150% compared to the control mix. The addition of nano-clay improves the shape stability of concrete [101]. Hybrid mixtures of VMA and nano-attapulgite clay have been used to regulate the rheology of concrete. A hybrid mixture of VMA and nano-clay was created using a magnetic stirrer. Test results showed that hybrid admixture sharply increased the yield stress, and 1% nano-attapulgite was observed to be the critical dosage of nano-clay for increasing yield stress [97]. Zhang et al. [102] observed that nano-clay and silica fume increase the buildability of concrete. The control mix provided a height of 7.2 cm, whereas the separate use of silica fume and nano-clay provided heights of 15.6 and 18 cm, respectively. The combined use of silica fume and nano-clay provided a printable height of 26 cm. Moeini et al. [168] added 0.5% nano-clay and 1% VMA separately in printable mixes to regulate rheology. Compressive strength showed that the addition of nano-clay made the mortar stiffer, the failure mode was brittle, whereas VMA led to a failure such as plastic deformation under compressive load. Lightweight foam concrete (LWFC) has low yield stress and high workability. Printing of lightweight foam concrete is difficult due to the low shape retention factor and lower yield stress. Nano-silica particles (2%, 3%) have been added into LWFC to increase its structuration rate. ICAR rheometer measurements have shown that static yield stress values for the control mix, mix with 2% and 3% nano-silica as 0.078, 0.224, and 0.386 kPa, respectively. Similarly, control mix and mixes with 2% and 3% nano-silica had exhibited dynamic yield stress values of 0.061, 0.120, and 0.291 kPa, respectively. More layers could be printed with LWFC containing nano-silica than reference LWFC mix [93]. Kruger et al. [36] observed that 1% nano-silica is the optimum dosage of nano-silica for increasing the thixotropy of concrete. Cheng et al. [44] reported an improvement in the thixotropic behavior of 3D-printable concrete with the addition of bentonite. Szostak and Golewski [169] added nano-calcium silicate hydrate (CSH) seeds in concrete and observed a reduction in setting time and a rapid increase in earlier strength. Nano-graphite platelets increase the thermal conductivity of cementitious materials and induce self-sensing capability in concrete. Chougan et al. [91] used nano-graphite platelets in printable geopolymer mixes and observed positive effects on shape retention of mixes after extrusion from the nozzle due to the attractive forces between nano-particles and their strong-sorbent properties. Additionally, nano-graphene platelets also enhanced the flexural properties of printed concrete. Chu et al. [170] developed a high strength fiber-reinforced mix design for concrete 3D printing by using nano-calcium carbonate and fibers. The increase in extrusion pressure due to the addition of nanoparticles and fibers was compensated by increasing the dosage of the superplasticizer. The addition of nanoparticles increased the shape retention, buildability, and compressive strength as well as interlayer bond strength of hardened concrete. The above research studies suggest that nanomaterials are helpful for regulating rheology to optimize printability, but the cost of nanomaterials is comparatively higher than other ingredients of concrete that can affect the total cost of 3D printing construction.

## 5. Anisotropy in Mechanical Properties of 3D-Printed Concrete

**Figure 6:**
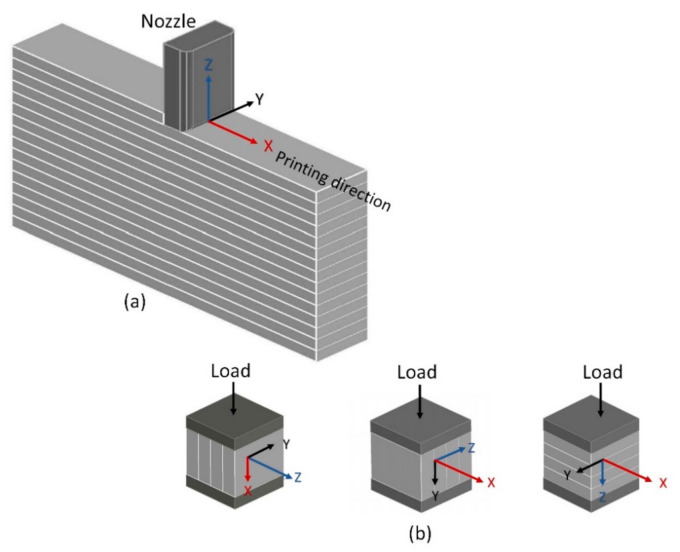
(**a**) Designation of directions *X*, *Y*, and *Z* axes, to evaluate the anisotropic mechanical behavior; (**b**) measurement of anisotropy in compressive strength.

3D-printed concrete has a layered structure due to the deposition of concrete in the shape of multiple layers to obtain a three-dimensional concrete element. There can be two types of interfaces in printed concrete elements. Extrusion of the succeeding layer over the top of the preceding layer creates a horizontal interface. If the succeeding layer is deposited adjacent to the preceding layer at the same level, then it will create a vertical interface between the two layers. Strength at the core part of the extruded concrete is higher than the strength at the horizontal or vertical interfaces of layers. Printed concrete elements have anisotropic mechanical behavior due to the dependency of performance upon the application of load direction with reference to the printing direction [174]. In the following sections, the anisotropic behavior of 3D-printed concrete in terms of mechanical properties has been quantified using the data obtained from the published literature. For this, orientations of extruded layers have been correlated with the printing direction, as shown in Figure 6a. Then compressive, flexure, and tensile strengths at three different orientations with respect to the printing direction have been collected from the various published research studies and compared with the corresponding strength values of casted concrete. It is worth mentioning here that the printed and casted specimens compared in this study have been prepared with the same concrete mix proportions. The only difference is the method of preparation that is casting versus extrusion.

### 5.1. Compressive Strength

**Figure 7:**
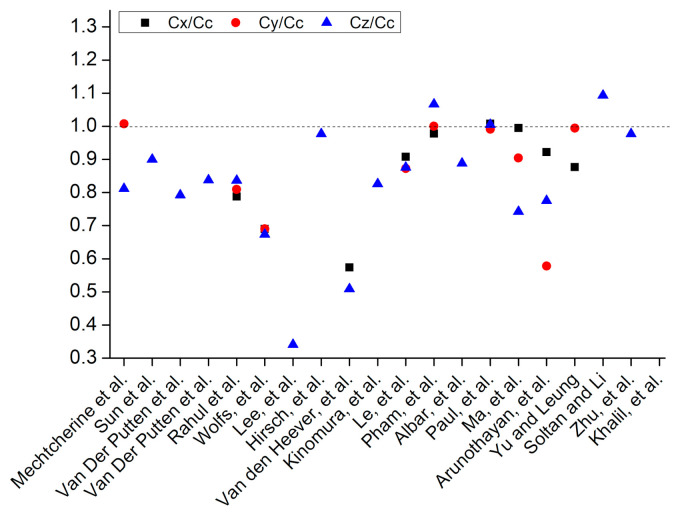
Anisotropy in compressive strength of 3D-printed concrete [1,49,61,92,127,135,165,175,176,177,178,179,180,181,182,183,184,185,186].

**Figure 8:**
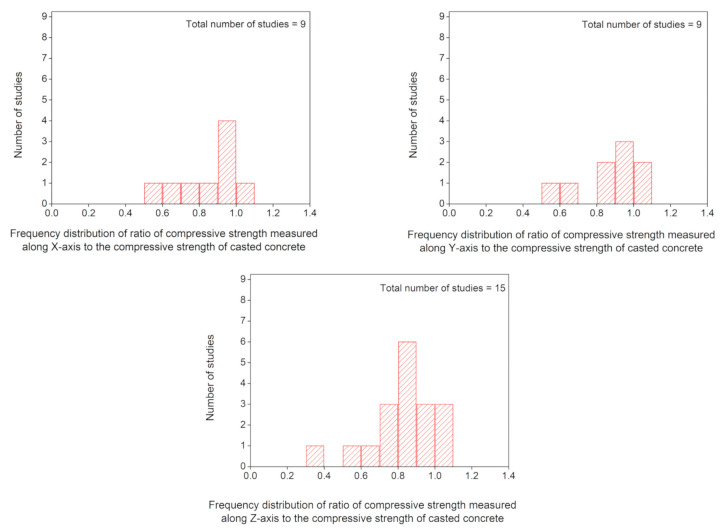
Frequency distribution of the anisotropy in compressive strength measured along *X*, *Y*, and *Z* axes.

Figure 6b shows the application of compressive load at three different orientations of extruded layers to identify the anisotropic behavior. Figure 7 represents the measured anisotropy in the compressive strength at these three orientations with respect to the printing direction of concrete where C_c_ shows the compressive strength of casted concrete and C_x_, C_y_, C_z_ show compressive strength measured along *X*, *Y*, and *Z* axes of printed concrete, respectively. For the measurement of anisotropy, compressive strength values along each axis have been divided by the compressive strength of casted concrete, so C_x_/C_c_, C_y_/C_c,_ and C_z_/C_c_ represent anisotropy in compressive strength along *X*, *Y*, and *Z* axes, respectively. A comparison of the compressive strength of printed concrete to that of casted concrete (Figure 7) shows that except for some exceptions [1,49,135,175], most of the researchers have reported a lower compressive strength for printed concrete than casted concrete. This implies that the printing process induces a reduction in the compressive strength of concrete. The data plotted in Figure 7 has been used in the calculations of Figure 8, which shows the frequency distribution of the ratio of compressive strength measured along the *X*, *Y*, and *Z* axes to the compressive strength of the casted concrete. Figure 8 shows that the most reported values of C_x_/C_c_, C_y_/C_c,_ and C_z_/C_c_ lie within the range of 0.9–1.0, 0.9–1.0, and 0.8–0.9, respectively, which suggests that lower compressive strength would be expected when the compressive load acts along *Z*-axis as compared to the *X*-axis and *Y*-axis. Pham et al. [175] and Sultan and Li [49] had reported higher compressive strength for the printed concrete (*Z*-axis) than the compressive strength of the corresponding casted concrete, but these researchers had used fiber-containing concrete for printing. Therefore, the order for anisotropic behavior in compressive strength of printed concrete (without fibers) could be C_x_/C_c_ = C_y_/C_c_ > C_z_/C_c_. From the structural performance perspective of a printed concrete structure, load applies along the *Z*-axis if the printed structure is a wall or a column, whereas, for a printed bench or stairs, load applies along the *Y*-axis. Hence, structural analysis of these printed concrete structures should be conducted while taking into account the application of loads with respect to the direction of printing, anisotropic behavior, and reduction in the compressive strength produced due to the printing process.

### 5.2. Flexural Strength

**Figure 9:**
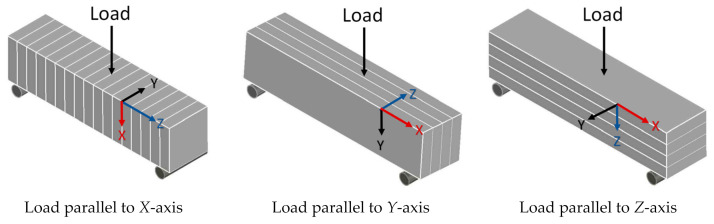
Designation of *X*, *Y*, and *Z* axes for the measurement of the anisotropy in flexural strength of printed concrete.

**Figure 10:**
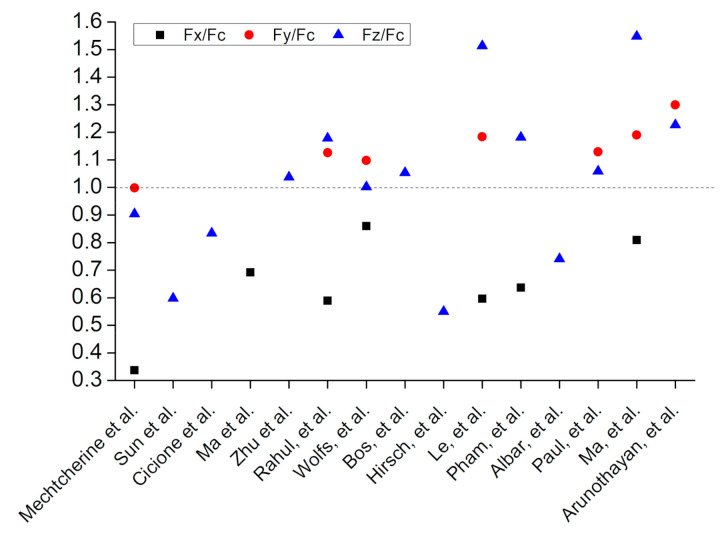
Anisotropy in flexural strength of 3D-printed concrete at different printing directions [1,61,125,127,135,142,151,165,175,178,179,180,183,184,187].

Figure 9 shows the application of the flexural load with respect to three different orientations of extruded concrete layers. Figure 10 shows the calculated anisotropy in flexural strength, where F_c_ shows flexural strength of casted concrete, F_x_, F_y_, and F_z_ show flexural strengths measured along the *X*, *Y*, and *Z* axes of printed concrete, respectively. A critical view of the data plotted in Figure 10 indicates two interesting pieces of evidence: first, the flexural strength of printed concrete along the *X*-axis has been reported lower than the flexural strength of casted concrete. Second, the flexural strength measured along the *X*-axis has also been reported lower than the flexural strength measured along the *Y* and *Z* axes [1,125,127,135,151,175,178,179,183,184]. This is due to the creation of tensile stresses between the extruded layers at the interface when flexural force applies along *X*-axis. The interface is the weakest point of printed concrete; therefore, it could not resist tensile stresses, and hence, flexural strength along the *X*-axis is lower. Figure 10 also indicates that flexural strength measured along the *Y*-axis has been observed to be higher than that of casted concrete [125,127,135,178,179,183] except Mechtcherine et al. [1]. Mixed reports are available for the flexural strength along the *Z*-axis. Some studies have reported it to be lower than the flexural strength of casted concrete [1,142,165,180,184] while others have observed opposite results [61,125,127,135,175,178,183,187]. It is notable here that fiber-containing printable mixes [125,127] have always exhibited improved flexural strength along the *Z*-axis than the corresponding flexural strength of casted concrete. The increase in flexural performance of fiber-reinforced printable mixes has been associated with the alignment of the fibers along the printing direction at the extruding nozzle [61]. Fibers prevent the microcracks development into macrocracks and stitch together cracked-printed concrete parts, consequently increasing the flexural strength [95,188]. Alignment of fibers with the printing direction increases in the case of using smaller-sized nozzles and higher dosage of fibers. Fibers close to the nozzle wall have more chances to be aligned with the printing direction than the fibers at the center of the nozzle [127,189].

**Figure 11:**
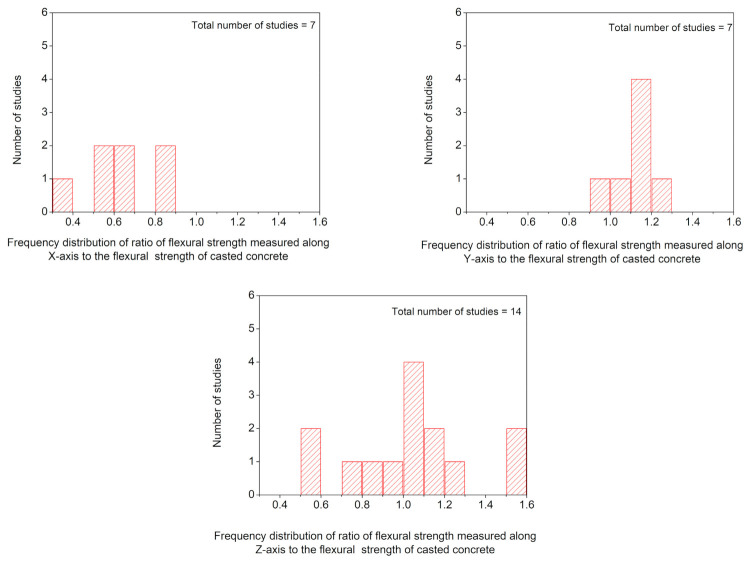
Frequency distribution of anisotropy in flexural strength measured along *X*, *Y*, and *Z* axes.

Figure 11 shows the frequency distribution of the ratio of flexural strength values measured at three different orientations to the flexural strength of casted concrete. Figure 11 shows that the most reported values of F_x_/F_c_, F_y_/F_c,_ and F_z_/F_c_ lie within the range of 0.5–0.7, 1.1–1.2, and 1.0–1.1, respectively, which suggests that higher flexural strength would be expected when the flexural load acts along *Y*-axis as compared to other two axes specifically *X*-axis. The order for anisotropic behavior in flexural strength of printed concrete (without fibers) could be F_y_/F_c_ > F_z_/F_c_ > F_x_/F_c_. Flexural strength is an important consideration for the digital construction of structural beams and bridge decks, and these structures should be designed while taking into account the anisotropic behavior in the flexural performance of printed concrete.

### 5.3. Tensile Strength

**Figure 12:**
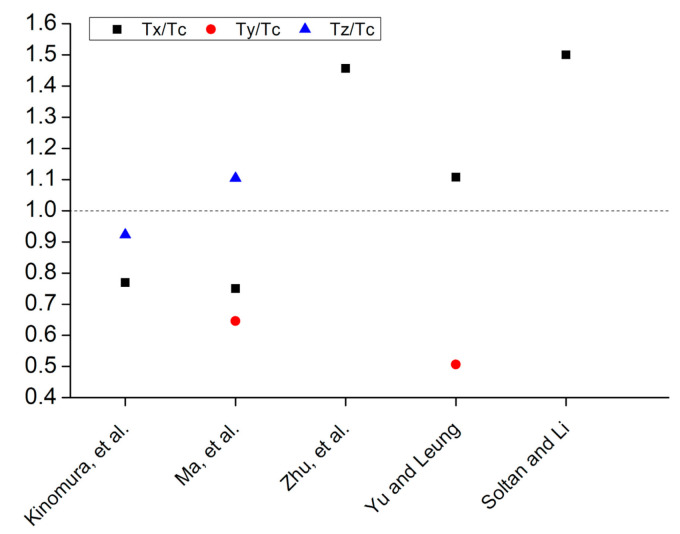
Anisotropy in tensile strength of 3D-printed concrete at different axes with respect to the printing direction [49,61,127,182,186].

Contrary to the compressive and flexural strengths of printed concrete, a few research studies have investigated the tensile strength of printed concrete at more than one axis. Figure 12 shows anisotropy in tensile strength measured at three different orientations of printed layers where T_c_ shows the tensile strength of casted concrete and T_x_, T_y_, T_z_ show the tensile strengths measured along the *X*, *Y*, and *Z* axes, respectively. It is worth mentioning here that most of these studies have used fiber-reinforced concrete for printing [49,61,127,186] except Kinomura et al. [182]. Additionally, these research studies have used different test methods for the measurement of tensile strength. Kinomura et al. [182] used splitting tensile strength test upon cylindrical specimens, Ma et al. [127] used splitting tensile strength test upon cubic specimens, Zhu et al. [61] and Yu and Leung [186] used dog-bone test specimens, and Soltan and Li [49] tested rectangular sections in the uniaxial test setup. Figure 12 shows that researchers have reported higher tensile strength along the *X*-axis for printed fiber-reinforced concrete than casted concrete [49,61,186]. Kinomura et al. observed lower tensile strength for printed concrete without fibers [182]. Based on the limited research studies, it can be suggested that printed fiber-reinforced concrete elements would have enhanced tensile strength along *X*-axis than the tensile strength of corresponding casted concrete due to the orientation of fibers along the printing direction. Whereas for printed concrete without fibers, reduced tensile strength is expected due to the imperfections induced due to the printing process. Further research is required to investigate the anisotropic behavior in tensile strength when measured along the *Y* and *Z* axes. Furthermore, concrete printing has been used to manufacture formwork for concrete construction and also for aesthetically appealing household items [190,191]. Tensile strengths are important for these structures and also for printed structural beams and bridge decks. Additionally, the lifting process of printed concrete structures (beam, column, shell) with a crane induces tensile loads in these structural elements. Anisotropic behavior in the tensile response of these printed structures should be considered for the above cases too.

### 5.4. Ratio of Flexural Strength to the Compressive Strength

**Figure 13:**
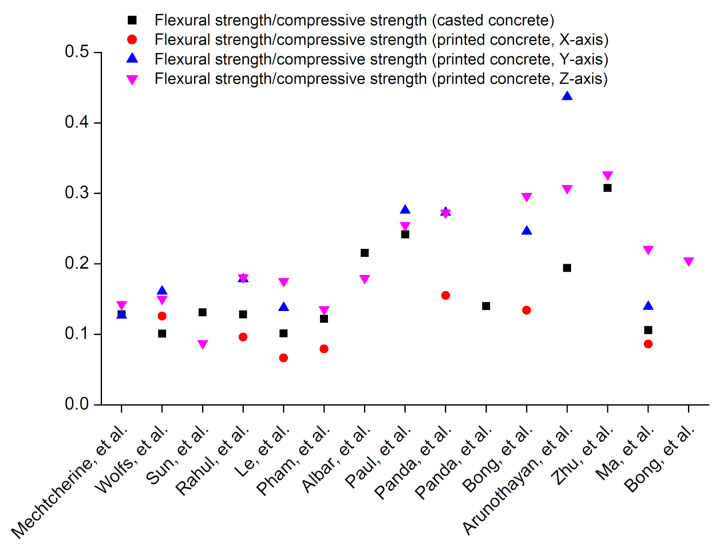
A comparison of the ratio of flexural strength to the compressive strength [1,61,126,127,135,165,175,178,179,183,184,185,192,193,194].

**Figure 14:**
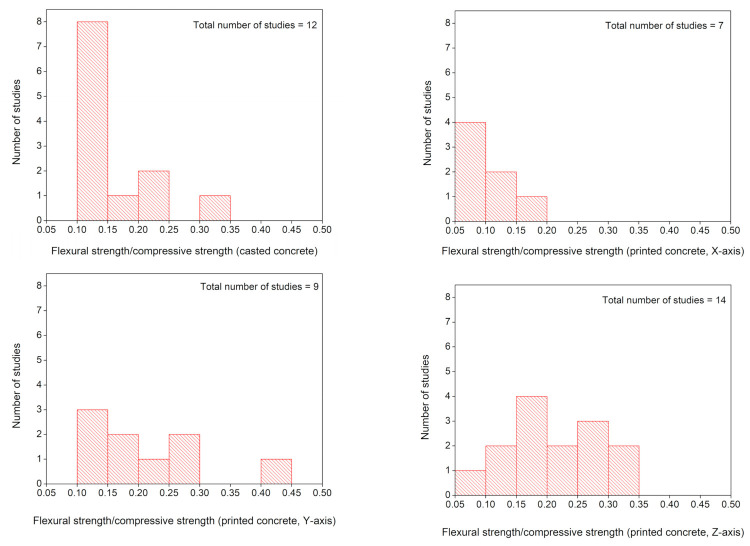
Frequency distribution of the ratio of flexural strength to the compressive strength in casted and printed concrete at different test directions.

In this review paper, a comparison of flexural strength with compressive strength has been conducted by dividing flexural strength measured along the three orientations (*X*, *Y*, and *Z* axes) to the corresponding compressive strengths. Figure 13 shows that in printed concrete elements, the results of the ratio of flexural strength to the compressive strength are dependent upon the applied load direction with respect to the extruded layers orientation, which indicates anisotropic behavior in this ratio of flexural to compressive strengths for printed concrete. For casted concrete, the ratio of flexure to compressive strengths is also plotted in Figure 13. Figure 14 shows the frequency distribution of the ratio of flexural strength to the compressive strength in printed and casted concrete. The ratio of flexural strength to the compressive strength lies within the range of 0.10–0.15 for casted concrete except for fiber-reinforced concrete mixes [61,135,185] as the inclusion of fibers significantly enhances flexural strength than compressive strength and consequently increase this ratio. For printed concrete, the ratio of flexural strength to the compressive strength along the *X*-axis lies within the range of 0.05–0.20. High variance is observed for the ratio of flexural strength to the compressive strength along the *Y* and *Z* axes, and the tendency of this ratio lies higher than 0.10, which indicates that printing layers act as laminated layers and increase the flexural strength along *Y* and *Z* axes.

## 6. Effect of Printing Process Parameters on the Mechanical Properties of 3D-Printed Concrete

Printing process parameters (time gap, printing speed, nozzle gap distance) influence the hardened behavior of 3D-printed concrete. The following subsections discuss the effect of these printing process parameters on the mechanical properties of printed concrete in the light of the previously published research papers.

### 6.1. Printing Time Gap

There is a unanimous understanding between the researchers that as the time gap between the layers increases, compressive, flexural, and tensile strengths of 3D-printed concrete decrease. Wolf et al. [179] tested the effect of various time gaps (15 s, 1 h, 4 h, 7 h, and 24 h) upon the flexural strength and noted that flexural strength reduced with the increase in the time gap between the layers. Chen et al. [195] measured the tensile bond strength between layers with print time gaps of 20 s, 1 min, and 10 min. Specimens with 20 s showed maximum bond strength. An increase in the time gap from 1 min to 20 min reduced the bond strength by 13% compared to the bond strength of casted specimens. The time gap between layers affects critical failure strength and failure mode of 3D-printed concrete. Wolf et al. [179] observed that printed specimens with a short time gap showed a flexural and splitting failure mode similar to the casted specimens where cracks originated from the bottom of the specimens and traveled haphazardly through the specimens, but as time increased, the crack traveled through the interface of the layers. Van Der Putten et al. [177] studied the effect of the time gap (0, 10, 30, and 60 min) upon the compressive and interlayer bond strengths and observed that increasing the time gap reduces the compressive strength and bond strength. Nerella et al. [77] and Panda et al. [193] observed reductions in flexural strength and tensile bond strength, respectively, with the increasing time gaps. Napolitano et al. [196] printed concrete with varying time gaps (0, 10, and 30 min) and measured the tensile strengths under varying strain rates. The 30 min time gap reduced the tensile strength by 58% compared to the 0 min time gap.

Panda et al. [56] associated the decrease in bond strength of printed concrete with the increasing time gap due to the differential structuration rate between two printed layers. As the time gap increases between layers, structuration occurs in the bottom layer and makes it stiff. When the successive layer is printed over the bottom layer, enough intermixing of layers does not occur, which reduces the bond strength. This process can be more prominent if rapid hardening cement is used in 3D printing [159]. Wolf et al. [179] tested the influence of two different scenarios upon strength. The tested scenarios were: (i) covering the bottom layer and (ii) exposing the bottom layer before printing the successive layer over it. Results showed that the exposure of the concrete substrate to the drying situation reduces flexural strength. Kieta et al. [74] exposed the concrete bottom layer in a wind tunnel and observed that the reduction in bond strength is due to the evaporation of water from the exposed surface of concrete during the printing time gap. Water evaporation creates dry substrate and leads to the reduced cement hydration and high porosity at the interface. A higher time gap leads to more water evaporation from the surface and consequently more reduction in bond strength. Van Der Putten et al. [177] studied surface moisture content on the surface of fresh-printed concrete. Most of the surface water evaporation occurred within the first 10 min. Sanjayan et al. [197] reported that in addition to the water evaporation, water bleeding from the bottom layer also affects bond strength between two layers.

The printing time gap affects bond strength between extruded layers significantly. Therefore, various researchers have attempted to compensate for weak interlayer bonds in the concrete printing process by proposing additional materials and treatments. Marchment et al. [133] applied cement paste between two layers to increase the effective bond area between layers. The application of the paste worked as a glue between two layers and enhanced the bond strength. A strong correlation between the effective bond area and the bond strength between layers was observed. Li et al. [198] developed a special mix design for application between printed layers to improve the interlayer bond strength for 3D-printed concrete. This mix contained calcium sulfoaluminate cement, cellulose fiber, silica sand, and limestone filler. Experimental results showed that this method significantly improved the bond strength between layers and allowed for an increased time gap between two layers. Wang et al. [199] attempted to improve interlayer bond strength by applying-polymer-modified mortars at the interface of the two layers. Shear and tensile strength were measured with a crossover test setup. Results showed that the shear and tensile bonding at the interface increased due to the electrostatic interaction between calcium ions of calcium-silicate-hydrate gel and epoxy resin present in polymer-modified mortar.

### 6.2. Printing Speed

Van Der Putten et al. [177] studied the effect of two different printing speeds (1.7 and 3 cm/s) upon the compressive strength and interlayer bond strength. The increase in printing speed reduced compressive strength and interlayer bond strength. Panda et al. [193] printed fly ash-based geopolymer concrete at three different printing speeds (7, 9, and 11 cm/s). Test results showed that an increase in printing speed reduces the width of the printed layers and can create microvoids. The difference in bond strength was indifferent at both printing speeds. Further investigation is required to identify the printing speed effect upon mechanical properties.

### 6.3. Nozzle Gap Distance

Wolf et al. [179] printed concrete with three different nozzle heights (0.80, 0.95, and 1.10 cm) using two different time gaps (15 s and 24 h). Test results did not show any clear influence of above nozzle gap distances upon flexural strength. Panda et al. [56] investigated the effect of two different nozzle heights (1.5 and 2 cm) on the bond strength of two different concrete mix proportions (control mix and a highly thixotropic mix). The influence of nozzle height was not detected in the control mix, but 2 cm nozzle height reduced bond strength in the highly thixotropic mix. In another study, Panda et al. [193] noted that nozzle height affects the quality of print and bond strength between layers. Their test results showed that 0.4, 0.2, and 0 cm nozzle standoff distances provided bond strengths of 1.5, 1.8, and 2.3 MPa, respectively, which indicate the positive effect of the reduced nozzle height upon the bond strength. Lee et al. [92] observed that nozzle height influences tensile bond strength between layers. Tensile bond strength values of concrete printed with a nozzle standoff distance of 2 cm were 70% of the tensile bond strength of the same concrete mix proportion printed with a nozzle standoff distance of 1 cm. Chen et al. [195] observed that variation of nozzle standoff distance from 0 to 0.5 and 1 cm decreases the bond strength, but the effect is insignificant. The above research results show that a reduced nozzle height is favorable for the mechanical properties of hardened printed concrete. Nozzle distance should be kept fixed throughout the printing process, but a layer printed with a mix proportion that has a lower shape retention factor would deform after extrusion. Printing of successive layers over it would further increase the deformation, and consequently, nozzle standoff distance would increase. Researchers in Coral Lab, Yonsei University, Republic of Korea, have developed a smart nozzle equipped with a depth sensor that detects the nozzle gap distance and adjusts the print head position to maintain the fixed height of nozzle standoff distance [200,201].

## 7. Reinforcement Strategies for Concrete 3D Printing

Traditional reinforcing techniques are not compatible with the concrete 3D-printing process [8,202]. Researchers have presented various alternative techniques for reinforcing extruded concrete which are briefly discussed below.

### 7.1. Cable Introduction at the Nozzle

This method introduces a continuous reinforcing cable into the extruding concrete at the nozzle. A reel on which a reinforcing cable is wound is attached to the print head, and the open end of the reinforcing cable is passed into the nozzle through a hole. Simultaneous extrusion of concrete and reinforcing cable through the nozzle winds off reinforcing cable from the reel attached to the print head and introduces it continuously into the concrete filament. Bos et al. [203] were the first to introduce this reinforcement strategy. Their pull-out test results showed that the bond strength of the cable with printed concrete was lower compared to its bond with casted concrete. Four-point bending test showed ductile failure behavior in cable-reinforced printed concrete accompanied with cable slip. Bending test results showed that the flexural response of the printed concrete can be improved with this reinforcement method. Similarly, the ductility of the printed concrete parallel to the printing direction can be improved by providing enough cable anchorage length. Li et al. [198] reinforced geopolymer concrete by this technique. Five different types of fibers (steel, nylon, carbon, aramid, and polyethylene micro cables) were tested. The use of steel micro cables produced maximum improvement in the mechanical properties, but shear strength was not increased with the introduction of the cables. The pull-out test showed that the anchorage length required for printed concrete is more than that for conventional concrete due to the reduced surface area and smooth surface of the reinforcing cable. Mechtcherine et al. [204] used mineral impregnated carbon fiber (MCF) for reinforcing concrete filament. MCF cable was introduced into the extruding layer at the nozzle, which enhanced the flexural strength of the printed concrete. Ducoullombier et al. [205] introduced co-extrusion of multiple continuous fibers and concrete through the nozzle simultaneously. Multiple yarns were continuously added to the concrete before printing, whereas the rheology of concrete was tuned such that its extrusion causes pultrusion of fibers in the extruded filament. Lim et al. [206] reinforced geopolymer concrete with the direct introduction of a continuous steel cable during extrusion from the nozzle. The four-point bending test showed that the flexural strength of the geopolymer increased by 290% due to cable reinforcement. Although cable introduction at the nozzle increases flexural strength and ductility of printed concrete, the interface between reinforcing cable and printed concrete has been reported to be porous [207]; therefore, this reinforcing technique needs further development.

### 7.2. Insertion of Reinforcing Elements into the Printed Concrete

Another approach is to first print a concrete layer and then insert reinforcing elements into the printed concrete with a device attached to the print head. Perrot et al. [208] reinforced printed concrete by inserting steel nails at different orientations, which increased the flexural reinforcement of printed concrete. Bester et al. [209] inserted steel fibers perpendicular to the interface of the printed layers. Fibers insertion increased the flexural strength and ductility of printed concrete. Geneidy et al. [210] proposed the simultaneous stapling of extruded layers with U-shaped reinforcement cables. A tool attached to the print head was designed for the simultaneous stapling of concrete layers, which effectively stapled extruded layers. Marchment and Sanjayan [211] inserted helical and deformed steel bars into the printed elements and measured the flexural strength under a three-point bending test. Deformed and helical steel bars increased flexural strength by 184% and 142%, respectively. Deformed bars had a better bond with concrete than helical bars, but failure mode in both cases was brittle due to the slippage of the reinforcement. In another study, Marchment and Sanjayan [212] inserted conventional deformed bars into the printed layers and observed that the bond between concrete and inserted bars was higher at the bottom layers than at the top layers. Hass and Bos [213] inserted screw-type reinforcement by simultaneous translational and rotational movement in the freshly printed concrete for reinforcement purposes. Pull-out and three-point bending tests were conducted to evaluate the effectiveness of this strategy. A suitable bond was observed between screw reinforcement when it was inserted in the earlier ages. These reinforcing approaches have the potential to increase the mechanical properties of concrete, but most of these methods are manual. Research is needed for the development of automated print heads for inserting reinforcing elements into the extruded concrete.

### 7.3. Mesh Reinforcement

Marchment and Sanjayan [214] reinforced concrete by embedding vertical steel mesh in the extruding concrete filament with a custom-designed nozzle. Mesh reinforcements were overlapped in the layers to produce a continuous reinforcement throughout the height of the printing wall. Experimental tests showed strong bond strength between mesh and concrete. Test specimens fractured due to the steel yielding instead of the steel mesh slip from the concrete. An increase in flexural strength up to 170–290% was achieved with embedded mesh reinforcement. Researchers at ETH Zurich invented mesh molding for the digital concrete construction of building walls. A robot manufactured a three-dimensional steel reinforcement mesh. Concrete was poured inside steel mesh to produce a load-bearing wall structure [215]. Wang et al. [141] placed a horizontal textile mesh between the layers of concrete for reinforcement. The three-point bending test showed that flexural strength increases as the number of layers of textile mesh increases. The use of three layers of textiles in the printed concrete increased its flexural strength equal to the casted concrete beam. Lin et al. [216] used polymeric mesh for reinforcing concrete. Compressive tests showed that interlayer polymeric mesh enhanced the ductility of printed concrete.

### 7.4. Printing over Conventional Bars

This method involves the horizontal placement of reinforcing bars upon freshly printed layers, followed by printing additional layers over them. Baz et al. printed concrete over the conventional reinforcing steel bar (8 mm) and measured the influence of concrete workability upon the bond strength between steel and printed concrete. Results showed that pull-out strength was not affected by the workability of concrete and the direction of printing [137]. However, in another study, Baz et al. observed that a high thixotropic printable mix produces a suitable bond between concrete and steel [217]. The placement of bars has been manual in these studies, which need to be automated.

### 7.5. Use of Printed Reinforcement

Mechtcherine [218] proposed 3D printing of steel reinforcement with a gas metal arc welding process. Test results showed that the ductility and bond strength of printed steel bars was similar to that of the conventional steel bars. Weger et al. [219] also observed that reinforcement with complex geometries can be produced using wire and arc additive manufacturing followed by selective paste intrusion to bond cementitious composite with the printed reinforcement. Katzer and Szatkiewicz [220] printed plastic formwork with the ribbed structures as a substitute for steel reinforcement of 3D-printed concrete. Tests showed that concrete makes a composite structure with printed plastic formwork after hardening and its flexural strength significantly increases. Adopting the use of printed reinforcement strategy would need two different setups, one for printing reinforcement and another one for extruding concrete, which may increase operational costs.

### 7.6. Fiber-Reinforced Printable Concrete Mix

The use of fiber-reinforced concrete and engineered cementitious composites (ECC) into 3D concrete printing is considered an alternative to the above reinforcing strategies. Such cementitious mixes do not need any additional automated setup at the extruding nozzle. Extruding of fiber-containing concrete mix aligns fibers parallel to the printing direction and enhances the mechanical performance. Panda et al. [192] added 0.25% and 1% short glass fibers in geopolymer concrete and tested influence upon compressive, flexural, and tensile strengths. Test results showed that the addition of fibers significantly increased the flexural and tensile strengths of concrete but did not affect the compressive strength. Hambach and Volkmer [221] used glass, basalt, and carbon fibers for the reinforcement of cement paste. The extrusion process aligned the fibers along the direction of printing and expressively enhanced the flexural strength. The flexural strength of the control specimen was around 11 MPa whereas the addition of the carbon fibers (1% vol.) increased flexural strength values up to 30 MPa. The effect of glass and basalt fibers upon flexural strength was insignificant. Ma et al. [127] observed that the use of 0.5% basalt fiber produces a concrete mix proportion with optimum printability performance and hardened mechanical properties. Basalt fibers resisted the conversion of microcracks into macrocracks due to the crack bridging effect under external loads. Chougan et al. [95] investigated the influence of poly-vinyl alcohol fibers on the mechanical properties of printed geopolymer concrete. Test results showed that the addition of 0.25% poly-vinyl alcohol fibers increased the compressive and flexural strength by 10% and 24%, respectively, compared to reference samples. The increase in mechanical properties is associated with the crack bridging effect of the fibers. Zhu et al. [61] developed a printable engineered cementitious composite by adding polyethylene fibers and compared it with conventionally casted ECC. Compressive, flexural, tensile strengths and tensile strain values, as well as crack saturation, increased with the amount of PE fibers in ECC. Ding et al. [188] used polyethylene fibers to reinforce concrete and observed that the addition of fibers increases the number of cracks and spacing between cracks. The post-peak behavior of printed concrete under flexural strength was increased with the increasing length of fibers. SEM observations showed that some fibers were pulled out of the cement matrix while a few fibers fractured under flexural loading. Natural fibers are eco-friendly products and are cheaper than man-made fibers. These sustainable products have the potential to be used as reinforcing agents for printed concrete. Future research is needed to investigate the addition of natural fibers on the rheology and printability performance of concrete as well as the structural performance of printed concrete [222]. The susceptibility of natural fibers to corrosion in the highly alkaline environment of printed concrete needs investigation too.

### 7.7. Post-Printed Reinforcement Strategies

In this method, the concrete structure is first printed into various designed segments on a flat surface, and then post-processing techniques such as assembling of segments, passing of reinforcement bars through the holes, post-tensioning, and grouting are used to reinforce the printed segments. Salet et al. [25] constructed a bicycle bridge by printing its components at the print facility of the Eindhoven University of Technology (TU/e) and then prestressed the sections at the site with the steel tendons passed through the holes of printed sections. Asprone et al. [27] manufactured reinforced concrete beams by first printing topologically optimized segments on a horizontal surface. After hardening, segments were assembled and reinforced with steel. Vantyghem et al. [28] constructed a concrete bridge girder by post-tensioning the printed segments with steel tendons and then added mortar grout in the cavities. Although this process seems more promising for constructing large load-bearing structures but post-processing steps involve labor involvement.

## 8. Microstructure of 3D-Printed Concrete

Three-dimensionally printed concrete consists of numerous extruded layers that are bonded to each other. The core part of layers has a dense microstructure, but the interface between layers has a porous structure [223]. Nerella et al. [77] examined printed concrete under scanning electron microscopy and detected voids at the interface and in the core part of the extruded concrete layers too, but the presence of voids was more in the interface region. Microcracks were present at the interface of layers due to the plastic and drying shrinkage of concrete. A part of these voids and cracks was filled with hydration products, carbonation, and self-healing products. Porosity in casted specimens is homogenously distributed, but the concentration of pores is higher at the interface in the printed specimens [195]. Chougan et al. [91] revealed that the addition of nano-graphite platelets arrests the microcracks development in printed concrete under flexural load. This crack bridging effect of nano-additives may be useful for blocking shrinkage cracks developed in hardened concrete too. Kruger et al. [224] observed an increased interlayer porosity as the time gap between horizontal layers increased. Micro-computed tomography examination showed 8.0% porosity at the vertical interface between layers and 7.7% porosity at the horizontal interface between layers, indicating higher porosity at the vertical interface between layers. X-ray computed tomography is a suitable technique for the investigation of the influence of concrete mix design and printing process parameters upon the porosity of printed concrete elements [225]. Lee et al. [226] investigated the porosity of printed concrete with X-ray computed tomography. Results showed that the porosity of printed concrete was higher at the interface. The location of the fracture plane under tensile bond strength in printed concrete was determined and compared with the distribution of porosity along with the specimen height. Results showed that the location of the fracture plane had no correlation with the porosity of the interface as it passed through the core in some specimens and through the interface in the rest of the specimens of the same batch. Panda et al. [56] noticed a porous interface when nozzle standoff distance was increased. Murcia et al. [227] observed more porosity at the vertical interface of the layers than the horizontal interface of layers and attributed lower porosity at the horizontal interface to the compaction induced by the weight of the top layers. Rheology affects the porosity at the interface of layers. Higher yield stress and thixotropy increase the stiffness of concrete, making it difficult for successive printing layer to adjust according to the rough surface of the bottom layer and thus enhance the density of the pores at the interface [228]. As 3D printing is performed in successive layers with a defined time gap, water evaporates from the top surface of the bottom layer to the exposed environment. Geng et al. [228] noted that the porosity of the horizontal interface significantly increases when water is allowed to evaporate from the top surface of the bottom layer, which consequently affects the interlayer bond strength. Bos et al. [229] have reported that pressing the bottom layer while printing the top layer increases the bond strength between layers and reduces the voids at the interface. Table 5 summarizes the porosity for printed and casted concrete. 

Another problem from the microstructural perspective of 3D-printed concrete is the presence of voids and connected porosity at the reinforcement-cement matrix interface [207,230]. This interface porosity can be minimized by first making a hole in the printed concrete, which is then filled with a grouting mortar, and then reinforcing bar is inserted into the hole [230]. Marchment and Sanjayan [231] coated the reinforcing bars with cement paste before insertion in printed concrete, which minimized the density of voids at the interface of bar and concrete and also improved the flexural strength. The above research studies elucidate that printed concrete is more porous than casted concrete and its porosity is dependent upon the concrete rheology, printing process parameters, and printing environment.

## 9. Durability of 3D-Printed Concrete

Researchers have focused more on tailoring the rheology, expediting the printing process of 3DCP as well as enhancement of mechanical properties than investigating the durability performance of printed concrete. A few research groups have studied the durability of 3D-printed concrete, and these research attempts are discussed in the subsections given below.

### 9.1. Chloride Attack

The connectivity of the pores at the interface increases the susceptibility of printed to the chloride attack [223]. Van Der Putten et al. [233] investigated chloride ions attack upon 3D-printed concrete using NT Build 443 [234] and compared it with casted concrete. Chloride ions ingress in printed concrete was higher than casted concrete. As the time gap increased between layers, the depth of chloride attack simultaneously increased. Blaakmeer and Lobo [235] studied chloride penetration and water sorptivity of printed concrete. The capillary water absorption of the outer layer was 0.1 kg/m^3^ higher than the inner bulk material due to its rough and porous nature. NT Build 492 test [236] showed that the chloride coefficient of concrete was 4 × 10^−12^ m^2^/s. Weger et al. [237] observed that increasing the time gap (0, 10, and 60 min) increases the rate of chloride attack. Van Der Putten et al. [238] also printed concrete with a time gap of 15 s with variable print speeds and evaluated the water sorption using neutron radiography. Water sorptivity was observed to decrease with the increase in the printing speed. Obtained radiographs showed no preferential water ingress through the interlayer when the print surface was exposed to the water.

### 9.2. Shrinkage Strains

Le et al. [183] observed that shrinkage was higher for printable concrete mix cured in a chamber (855 μm) with a relative humidity of 60%, temperature of 20 °C than its shrinkage strains cured in water (175 μm). Moelich et al. [239] observed higher plastic shrinkage strain in printed concrete due to the absence of formwork, lower sand-binder ratio, and higher amount of fine content in printable concrete than conventional concrete. Federowicz et al. [240] investigated the effect of adding shrinkage reducing admixture and foil insulation upon the shrinkage strains in printed concrete. The addition of shrinkage reducing admixture as 2% weight of cement reduced the strains by 7%, whereas 4% addition reduced the strains by 23%. External curing reduced strains by 80%, which is a more effective method, but it hinders the continuous printing of concrete. Moelich et al. [239] observed that most of the shrinkage cracks occur within the first two hours after printing. The provision of shrinkage restraining reinforcement increases the cracks’ density. The authors also reported slip between extruded layers due to differential shrinkage strains between layers [239]. Three-dimensionally printed concrete is more susceptible to shrinkage cracks due to the higher amount of binder used in the printable mixes. Additionally, shrinkage of 3DCP is also dependent upon the curing environment. Post-printed curing environments should be adopted for reducing shrinkage strains in printed concrete.

### 9.3. Freeze-Thaw Attack

Assaad et al. [241] studied the deterioration of printable concrete under a freeze-thaw attack environment and added air-entraining admixture and styrene-butadiene rubber to know its effects on the frost attack resistance of printed concrete. Frost attack reduced the compressive and flexural strengths of printed concrete, but the addition of air-entraining agents reduced the rate of structural damage. Compared to other mechanical properties, interlayer bond strength was more damaged. The improvement in the frost resistance in the presence of air-entraining agents was due to the presence of additional voids, whereas the addition of latex increased the flexibility of concrete, which compensated for the damages due to frost attack. Air-entraining agents are intentionally added to the concrete to increase its resistance to freeze-thaw attacks in cold regions [242]. Das et al. [243] observed that pumping of concrete decreases void diameters and spacing factors that were purposely added in concrete for resisting freeze and thaw attacks.

### 9.4. Fire Attack

The heating of 3D-printed concrete to elevated temperatures reduces its mechanical and microstructural performance, similar to casted concrete. Cicione et al. [142] compared the fire resistance of 3D-printed concrete with conventional concrete. Casted and printed specimens were exposed to high heat flux (50–60 kW/m^2^) until the temperature reached 300 °C. The fire resistance performance of printed concrete was similar to the casted concrete except that heating caused spalling in the casted concrete, but it caused the separation of layers at the interface of printed concrete. The better resistance of printed concrete to spalling can be attributed to its connected porosity and higher permeability compared to the casted concrete. D’Hondt et al. [244] exposed printed concrete to elevated temperatures (120, 250, 400, and 600 °C) and measured its residual mechanical properties. Test results showed conservation of compressive and flexural strengths, stiffness, and isotropic characteristics of printed concrete. Kruger et al. [245] investigated the thermal performance of 3D-printed concrete elements. Delamination of printed layers occurred at elevated temperatures contrary to the spalling that occurs in casted-high strength concrete. The addition of steel fibers perpendicular to the interface produced ductile behavior after the thermal attack. If the heating rate is high, then 3D-printed concrete can explosively spall. However, the use of polyethylene (PE) fibers in 3D-printed concrete reduce the risk of spalling as it melts around 200 °C and evaporates upon further heating. Evaporation of PE fibers creates microcracks through which water vapors can release and spalling risk is mitigated [246].

### 9.5. Research Needs for Durability Performance of Printed Concrete

**Figure 15:**
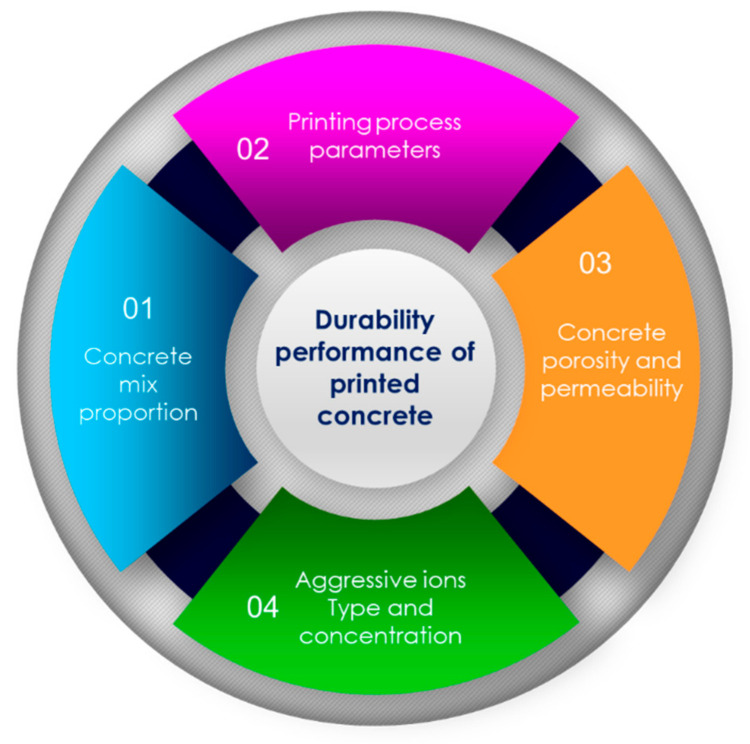
Parameters influencing the durability performance of 3D-printed concrete.

Additional in-depth investigations are required for measuring the durability performance of printed concrete in aggressive environmental conditions. The durability behavior of 3D-printed concrete would be different from conventional concrete due to the use of different mix proportions, high dosage of chemical admixtures, and layer-by-layer construction methods. Figure 15 shows the four main influential parameters (mix design, printing process parameters, transport properties of hardened printed concrete, and surrounding environment of printed concrete at the site), which would define the durability behavior of printed concrete. Binder type, water-binder ratio, binder-aggregate ratio, the dosage of chemical admixtures such as superplasticizers, retarders, viscosity modifying admixtures make the material part of the printed concrete, which needs to maintain the integrity of printed concrete under aggressive environmental conditions. Pore size distribution, connectivity of pores, the porosity of bulk layer, vertical and horizontal interfaces, and concrete permeability define the rate of the ingress of hostile ions inside the printed concrete, whereas these properties are dependent upon the printing process parameters such as nozzle height, printing time gap, and printing speed. Exposure environment at the site of printed concrete such as sulfate, chloride, carbonation, or leaching attack conditions defines the degradation mechanism and consequent effect on the mechanical and microstructural properties. Future research should evaluate the durability performance in view of these four parameters.

## 10. Conclusions and Future Research Needs

This paper critically reviews the latest research on the emerging 3D concrete printing from the standpoint of concrete materials. Unique rheological requirements of the concrete printing method, compatible concrete mix designs for printing, and the effect of eco-friendly binders, aggregates, chemical admixtures, and nanomaterials on the concrete rheology are discussed to help engineers and researchers recognize concrete printing procedures and develop their own mix proportions for this process. This paper gives special attention to the mechanical properties of 3D-printed concrete. It evaluates the anisotropy in compressive, flexural, tensile strengths and the effect of printing process parameters upon mechanical properties. Additionally, this paper also covers the latest research attempts aimed at improving the hardened properties of printed concrete and also discusses the durability performance of 3D-printed concrete in aggressive environments. The following concluding points and research needs are identified from this review work:

- Consensus on a single geometrical model for the measurement of the buildability of a concrete mix should be developed among researchers and industry practitioners. A standard geometrical model with defined layers width and height, number of layers, radius of curves, printing time gap, nozzle standoff distance, and travel speed should be developed so that buildability test measurements of different concrete types as well as different research groups could be easily compared and transmitted;
- Most of the test methods for the measurement of concrete extrudability and print quality are empirical and manual. These methods rely on human judgment. Inline test methods for calculating shape retention as well as the measurement of print quality in terms of the number of voids per unit length are required. The pumpability of 3D-printable concrete is less discussed in the literature. Lab-scale printers have used small to medium-sized pumps to transport concrete over shorter distances. In commercial projects, concrete would be pumped to larger distances. Investigation of the changes in concrete rheology while pumping it to larger distances and then extruding it through a contracting nozzle needs detailed investigation;
- Eco-friendly binders (silica fume, metakaolin, fly ashes), nanoparticles (nano-silica, nano-attapulgite clay), and chemical additives are very useful for tuning the rheology of concrete according to the requirements of the printing process;
- Compressive strength of printed concrete would be lower than the compressive strength of the corresponding casted concrete mix. The expected order for anisotropic behavior in compressive strength of printed concrete with respect to the casted concrete could be C_x_/C_c_ = C_y_/C_c_ > C_z_/C_c_. In other words, higher compressive strength is anticipated when load applies along *X* or *Y* axes compared to load application along *Z*-axis. The above order of compressive strength is not valid for fiber-containing concrete mixes;
- Flexural strength of printed concrete along the *Y*-axis could be higher than the flexural strength of corresponding casted concrete, but flexural strength along the *X*-axis is anticipated to be poor than the flexural strength of casted concrete. The order for anisotropic in flexural strength of printed concrete could be F_y_/F_c_ > F_z_/F_c_ > F_x_/F_c_. Higher flexural strength is expected when load applies along *Y*-axis, followed by *Z* and *X* axes (except fiber-containing mixes);
- Inferior tensile strength could be exhibited by printed concrete compared to corresponding casted concrete. However, in the case of using fiber-containing mixes, higher tensile strength is expected for printed concrete when tensile load applies parallel to the *X*-axis due to the alignment of fibers along the direction of printing;
- Flexural strength along the *X*-axis of printed concrete could be 5–10% of its compressive strength along the *X*-axis. For *Y*-axis, flexural strength would be more than 10% of the corresponding compressive strength. Better performance is expected along the *Z*-axis, where flexural strength would be more than 15% of corresponding compressive strength. Our literature survey also showed that for casted printable concrete mixes (without fibers), flexural strength mostly lies within the range of 10–15% of its compressive strength;
- Impact resistance and seismic performance of printed concrete have not been studied yet. These properties need to be studied for printed structures intended for military and industrial purposes or for construction at a seismic zone. Printed concrete is expected to exhibit a different seismic response than conventional casted concrete due to its anisotropic mechanical properties, which need detailed scientific investigation;
- Among the printing process parameters, the time gap is the more influential parameter to influence the mechanical properties of printed concrete especially interlayer bond strength. Variation of concrete rheology affects interlayer bond strength as well as the bond between steel and concrete;
- Research is needed for integrating reinforcement provision strategies as a part of the automation system. Additionally, reinforcement requirements for resisting shear, flexural, torsional stresses, and impact loads in 3D-printed concrete structures need investigation;
- Bond strength between printed concrete and reinforcement is reduced as compared to bond strength between casted concrete and reinforcing steel. The porous microstructure can develop at the interface of reinforcements and 3D-printed concrete. Densifying this interface as well as improving the bond strength between printed concrete and reinforcement is required;
- Porosity is high at the interface of layers, and at the reinforcement-concrete interface, pores at these interfaces can be connected, which can increase the permeability of printed concrete for aggressive ions. Reducing the porosity of printed concrete to improve its impermeability is a research issue. The higher content of binder used in concrete printing and the absence of the proper curing environment can exaggerate the shrinkage cracks, which can negatively affect the durability performance of concrete. The influence of rheology, printing process parameters, porosity, and shrinkage cracks on the durability performance of printed concrete in terms of alkali-silica reaction, delayed ettringite formation, sulfate, chloride, frost attacks, carbonation, and steel corrosion need investigation;
- Reinforcement attempts such as the introduction of the steel cable into the concrete filament at the printer nozzle and insertion/stapling of steel in printed concrete creates pores around the steel. These reinforcement methods need further development to simultaneously reinforce the concrete and create a dense interface with the printed concrete;
- Existing durability test methods are designed for casted concrete that has isotropic properties. On the contrary, printed concrete has anisotropic porosity properties. Research is required for the transport mode of aggressive ions into printed concrete and the development of new test methods for the durability behavior of printed concrete. Performance-based standards should be developed to design and print durable 3D-printed concrete structures.

## Figures and Tables

**Table 1:** Static yield stress values reported in literature for different printable concrete inks.

Concrete Ink Type	Reference	Testing Apparatus	Static Yield Stress (kPa)
Cement-based mortar	Perrot et al. [38]	Anton Paal Rheolab rheometer	4
Cement-SCM * blended mortar	Le et al. [40]	Shear vane test	0.3−0.9
	Rahul et al. [57]	Shear vane test	1.5−2.5
	Kruger et al. [36]	ICAR rheometer	2.7−3.9
	Kruger et al. [55]	ICAR rheometer	1.9
	Papachristoforou et al. [52]	ICAR rheometer	0.5−1.8
	Weng et al. [29]	Viskomat XL	3.3
	Panda et al. [56]	Anton Par MCR 102 rotational rheometer	3.2−6.8
	Moeini et al. [66]	Anton Paar MCR 302 rheometer	0.2−0.7
Geopolymer mortar	Panda and Tan [53]	Anton Par MCR 102 rotational rheometer	0.4−1
Cement paste	Chen et al. [54]	Rotational rheometer	0.2−0.7

* SCM: supplementary cementitious materials.

**Table 2:** Dynamic yield stress and plastic viscosity values reported in literature for different concrete inks.

Concrete Ink Type	Reference	Testing Apparatus	Dynamic Yield Stress (kPa)	Plastic Viscosity (Pa·s)
Cement-SCM blended mortar	Moeini et al. [66]	Anton Paar MCR 302 rheometer	0.1	1.9
	Zhang et al. [60]	-	0.1–0.2	3.5–4.1
	Jayathilakage et al. [67]	Rotational rheometer	1.2–1.8	24.2–47.1
Printable ECC *	Zhu et al. [61]	Brookfield RST-SST rheometer	0.2–0.5	3.7–11.7
Cement paste	Nair et al. [62]	Dynamic shear rheometer	0.1–0.3	1.6–4.2
	Chen et al. [54]	Rotational rheometer	0.5–0.6	2.4–2.6
	Chen et al. [58]		0.5–0.7	2.4–2.9
	Chen et al. [44]		0.6–0.7	2.2–3.4

* ECC: engineered cementitious composites.

**Table 3:** Representative mix designs for 3D concrete printing.

Concrete Mix Type	Reference	Binder	Water-Binder Ratio	Sand-Binder Ratio	Sand Size	Admixture (% Wob)	PP Fibers, Otherwise, as Stated (% Wob)
Cement-based mix	Perrot et al. [38]	Portland cement	0.41	1	0–0.1 mm	HRWRA = 0.3	-
	Van Der Putten et al. [129]	Portland cement	0.36	2	0–2 mm	HRWRA = 0.15	-
	Xu et al. [130]	Portland cement	0.35	1	0–1.2 mm	HRWRA = 0.1, Cellulose ether = 0.05	-
	Schröfl et al. [131]	Portland cement	0.42	1.7	0–2 mm	HRWRA = 0.7	-
	Ding et al. [132]	Portland cement	0.39	1	0–1.2 mm	HRWRA = 0.18, VMA = 0.12	
	Marchment et al. [133]	Portland cement	0.36	1.5	0–2 mm	-	-
	Khalil et al. [134]	Portland cement	0.35	1.18	0–2 mm	HRWRA = 0.2, calcium sulfoaluminate = 7	-
Cement-SCM blended mix	Lee et al. [92]	Portland cement, fly ash, silica fume (0.70:0.20:0.10)	0.28	1.38	0.16–0.2 mm	HRWRA =1, VMA = 0.2	-
	Le et al. [40]	Portland cement, fly ash, silica fume (0.70:0.20:0.10)	0.28	1.5	0–2 mm	1	0.19
	Nerella et al. [59]	Portland cement, fly ash, silica fume (0.26:0.26:0.48)	0.42	1.54	0–2 mm	HRWRA = 2–3	-
	Zhang et al. [60]	Portland cement, silica fume, nano-clay (0.96:0.02:0.02)	0.35	1	0–1 mm	HRWRA = 0.26, VMA = 0.01, nano-clay = 2	-
Cement-SCM blended mix	Paul et al. [135]	Portland cement, fly ash, silica fume (0.41:0.39:0.20)	0.4	1.7	0–1 mm	HRWRA = 1	-
	Tay et al. [81]	Portland cement, fly ash, silica fume (0.70:0.20:0.10)	0.49	1.7	0–2.36 mm	-	-
	Rahul and Santhanam [136]	Portland cement, fly ash (0.80:0.20)	0.32	1.5	-	HRWRA = 0.08, VMA = 0.25	-
	Baz et al. [137]	Portland cement, silica fume (0.90:0.10)	0.4	1.25	0–1.5 mm	HRWRA = 0.26–0.40	-
	Mechtcherine et al. [1]	Portland cement, fly ash, silica fume (0.59:0.23:0.17)	0.3	2.5	0.06–8 mm	HRWRA = 0.84	-
	Tao et al. [138]	Portland cement, limestone powder (0.75:0.25)	0.36	1.55	0.1–2 mm	HRWRA = 0.5, VMA = 0.2	-
	Suntharalingam et al. [139]	Portland cement, slag, fly ash (0.55:0.3:0.15)	0.31	1.18	-	HRWRA = 1.2, VMA = 0.6	-
	Xu et al. [140]	Portland cement, fly ash (0.44:0.56)	0.42	0.374	-	HRWRA = 0.08, VMA = 0.03	-
	Wang et al. [141]	Portland cement, fly ash, silica fume (0.70:0.20:0.10)	0.26	1.5	-	HRWRA = 1, Retarder = 0.5,	0.14
	Cicione et al. [142]	Portland cement, fly ash, silica fume (0.70:0.20:0.10)	0.45	1.41	0–4.75 mm	HRWRA = 0.7	-
	Rahul et al. [57]	Portland cement, fly ash, silica fume (0.70:0.20:0.10)	0.32	1.5	0–2 mm	HRWRA = 0.17	0.2
	Kazemian et al. [101]	OPC type II, silica fume (0.90:0.10)	0.43	2.3	0–2.36 mm	HRWRA = 0.15, nano-clay = 0.3	-
	Moeini et al. [66]	Portland cement, fly ash, silica fume (0.70:0.25:0.05)	0.35	0.75	0–1 mm	HRWRA = 0.3, clay = 0.5	-
Geopolymer concrete mix	Panda and Tan [53]	Fly ash, Slag, silica fume, potassium silicate,		1.5	0–2 mm	Nano-clay = 1.2, fiber = 0.25	-
	Bong et al. [143]	Fly ash, slag (0.50:0.50)	0.36	1.5	-	Retarder = 0.5, alkali activator = 10	
Fiber-reinforced composite	Ma et al. [127]	Portland cement, fly ash, silica fume (0.70:0.20:0.10)	0.26	1.19	average size = 0.39 mm	HRWRA = 1.8	Basalt fiber, 0.5
	Arunothayan et al. [125]	Portland cement, silica fume (0.70:0.30)	0.16	1	-	HRWRA = 1.5, VMA = 0.1	Steel fibers, 2% by volume
Engineered cementitious composite (ECC)	Zhu et al. [61]	Portland cement, sulfoaluminate cement, fly ash (0.40:0.03:0.57)	0.28	0.40	0–0.3 mm	HRWRA = 1.2, VMA = 0.1	Polyethylene fiber, 2% by volume
	Bao et al. [128]	Portland cement, calcium aluminate cement, Fly ash, (0.30:0.02:0.68)	0.25	0.38	-	Nano-clay = 0.3, VMA = 0.3, HRWRA = 0.9, nano-TiO2 =5	PVA fiber, 2%
Underwater concrete	Mazhoud et al. [118]	Portland cement and limestone (0.65:0.35)	0.38	1	0–2 mm	HRWRA = 0.5%,1%,1.5%, 3% Anti-wash agent = 0.5, 1.1, 1.5	-
Cement paste-based ink	Chen et al. [54]	Calcium sulfoaluminate cement, metakaolin (0.97:0.03)	0.35	Cement paste		Superplasticizer = 0.3, VMA = 0.4, retarder = 0.15	-
	Manikandan et al. [46]	Cement type II, silica fume (0.975:0.025)	0.3	Cement paste		1.5	-
	Moini et al. [144]	Portland cement	0.26	Cement paste		HRWRA = 0.4, VMA = 1.2	-

HRWRA = high-range water reducing agent, VMA = viscosity modifying agent, PP = polypropylene fibers, wob = weight of binder.

**Table 4:** Effect of ingredients upon the fresh properties of 3D-printable concrete.

	Workability	Yield Stress	Plastic Viscosity	Setting Time	Extrusion Pressure	Thixotropy	Green Strength	Shape Stability	Buildability	Print Quality	Reference
Metakaolin	↓			↓	↑	↑	↑	↑			[54,145]
Silica fume	↓	↑				↑	↑		↑	↑	[101,102,146]
Rice husk ash	↓	↑					↑				[148]
Municipal solid waste incinerated fly ash	↓	↑		↓			↑		↑		[41]
Fly ash	↑			↑							[171]
Limestone		↓		↓	↓			↑	↑		[86,145,147]
Mine tailings	↑								↓		[151]
Accelerator				↓					↑		[134,161]
Retarder				↑							[162]
Superplasticizer	↑	↓				↓					[163]
VMA			↑	↓	↑	↑		↑			[49,90,164]
Nano-attapulgite clay		↑				↑		↑	↑		[95,96,97,98]
Nano-CSH				↓			↑				[169]
Nano-silica		↑		↓		↑	↑		↑		[36,93,94]
Nano-calcium carbonate					↑		↑	↑	↑		[7]
Nano-graphite platelets								↑	↑		[91]
Bentonite						↑					[44]
Air-entraining admixture		↓					↓				[172]
Recycled glass cutlets		↓	↑						↓		[173]
Poly-vinyl alcohol fibers		↑						↑	↑		[95]
Expanded thermoplastic microspheres			↑					↑	↑		[153]

**Table 5:** Porosity of 3D-printed concrete versus casted concrete.

Study	Concrete Type	Test Method	Casted Concrete	Printed Concrete
			Porosity (%)	Porosity (%)
[178]	OPC-SCM blended concrete	Vacuum saturation method	9.7	Core part = 9.12, horizontal interface = 11.0, vertical interface = 11.2
[224]	OPC-SCM blended concrete	X-ray computed tomography	6.8	horizontal interface = 7.7, vertical interface = 8.0
[185]	Ultra-high-performance fiber-reinforced concrete	ASTM C20 [232]	10.3	10.9

## Data Availability

Data sharing not applicable.
